# Rapid Oxygen Atom Transfer at a Catalysis-Relevant Ni(I)–Alkyl Complex with N_2_O

**DOI:** 10.1021/jacs.5c03351

**Published:** 2025-05-30

**Authors:** Ana Mateos-Calbet, Paolo Cleto Bruzzese, Markella Aliki Mermigki, Alexander Schnegg, Dimitrios A. Pantazis, Josep Cornella

**Affiliations:** † 28314 Max-Planck-Institut für Kohlenforschung, Kaiser-Wilhelm-Platz 1, Mülheim an der Ruhr 45470, Germany; ‡ 28313 Max-Planck-Institut für Chemische Energiekonversion, Stiftstrasse 34−36, Mülheim an der Ruhr 45470, Germany

## Abstract

To improve our understanding of Ni-catalyzed alcohol formation with N_2_O, a catalytically relevant Ni­(I)–alkyl was synthesized and characterized (EPR, NMR). The complex, prepared via comproportionation and transmetalation, reacted with N_2_O at ambient temperature within 1 min, producing alcohol and N_2_. This contrasts sharply with slower reactions of (bpy)­NiEt_2_ (12 h) and catalytic systems (22 h). Theoretical studies suggest a stepwise organometallic Baeyer–Villiger mechanism via a Ni­(II)–oxyl intermediate. The high reactivity of Ni­(I)–alkyl challenges the notion of N_2_O’s inertness, highlighting its potential as a versatile oxidant in synthesis.

## Graphical abstract

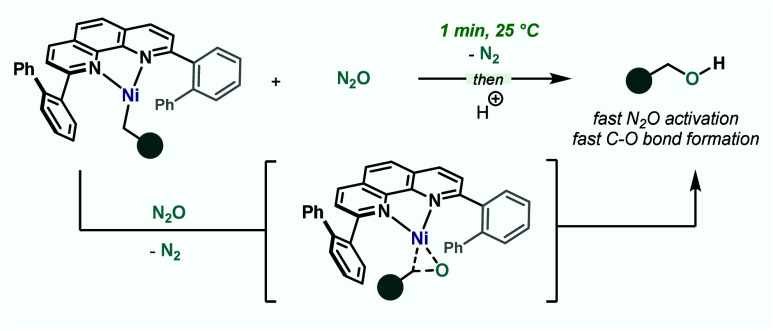

Nitrous oxide (N_2_O) is one of the most potent greenhouse gases in the atmosphere, and its concentration has been rising due to increased human activity.^1^ Despite its environmental implications, N_2_O is a potent oxygen atom transfer (OAT) reagent, releasing benign N_2_ as the sole byproduct upon N–O cleavage.^2^ Although inert, its activation has been explored with various metal complexes, leading to the traditional metal–oxo (M=O) reactivity (Figure 1A, left).^2a,3^ However, a pioneering work by Hillhouse and co-workers demonstrated that another reactivity paradigm beyond the canonical metal–oxo was also feasible: a process reminiscent to the Baeyer–Villiger reaction that leads to the O atom insertion into the M–C bond (Figure 1A, right).^4,5^ Such organometallic Baeyer—Villiger (OMBV) reactivity with N_2_O was initially observed with early transition metal complexes [e.g., Hf­(IV)], and later reported for the late transition metal Ni (Figure 1A, bottom).^4a,6^ Based on these stoichiometric precedents, our group initiated a program toward the development of Ni-catalyzed OAT reactions for the synthesis of alcohols using N_2_O.^7^ Of particular relevance is an unprecedented Ni-catalyzed formation of aliphatic alcohols, where a C­(sp^3^)–O bond is formed at room temperature under reductive conditions.^7b^ It is important to mention that similar to the stoichiometric precedents with Ni (Figure 1A, bottom), the catalytic reaction also requires prolonged reaction times (20–40 h), which has generally been attributed to the commonly challenging N_2_O activation by the metal center (Figure 1B). Based on the reductive conditions of the catalytic reaction, we imagined that a Ni­(I)–alkyl would be a viable intermediate, which could lead to an OAT to forge the alcohol (Figure 1B). Yet, differently than in Hillhouse’s examples, the intermediate postulated is a Ni­(I), and evidence for this OMBV-type process at such low-valent species has not been reported. Therefore, it would be of great interest to study whether a Ni­(I)–C­(sp^3^) interacts with and activates N_2_O, and whether it is able to form a C–O bond at room temperature. In this work, we provide experimental evidence for an *extremely fast reaction (<1 min)* at 25 °C between N_2_O and a novel Ni­(I)–alkyl complex bearing a catalysis-relevant phenanthroline ligand, releasing N_2_ and alcohol upon acidic work up (Figure 1C). A combined experimental and theoretical analysis of the system provides evidence for an energetically facile activation of N_2_O through N–O cleavage followed by an organometallic Baeyer–Villiger-type process, which has no precedents in the literature for low-valent Ni­(I).

**Figure 1:**
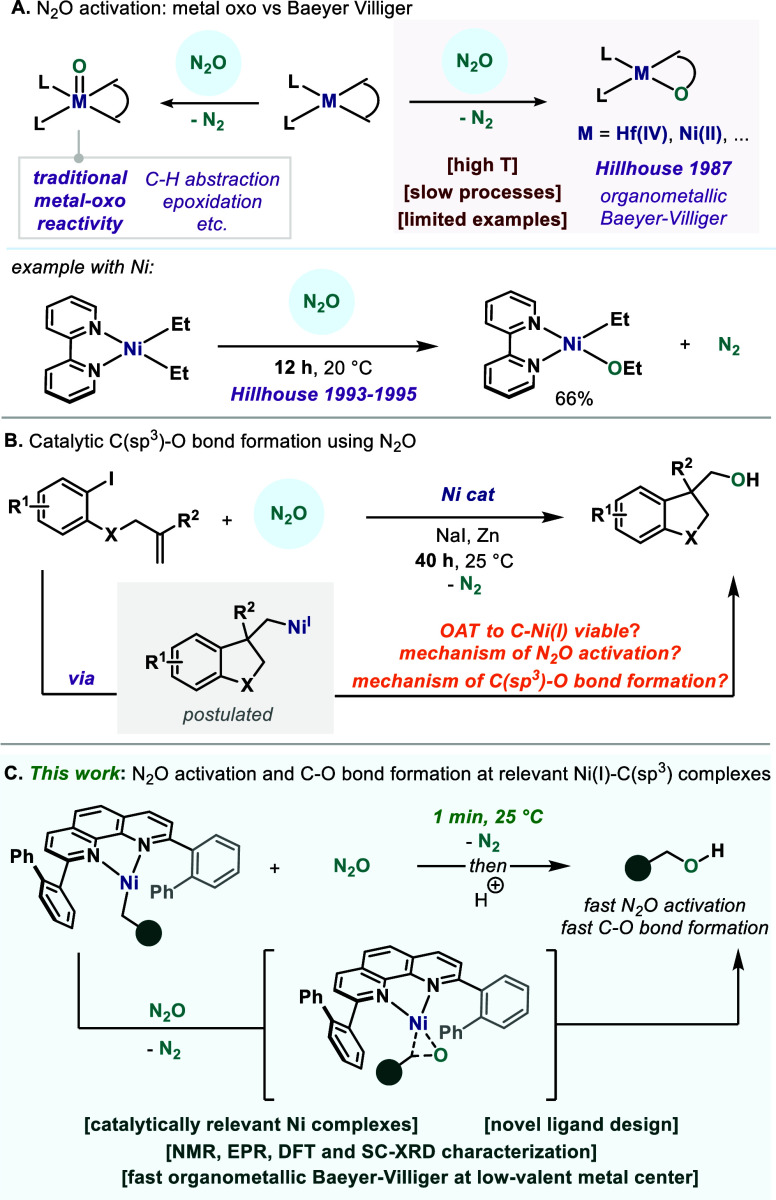
State of the art reactivity of N_2_O with Ni complexes and application in catalysis.

Initially, we attempted the synthesis of a Ni complex bearing the catalytically competent **L1** ligand. However, solubility challenges, complex speciation, and instability of its low-valent analogues hindered their isolation, making it an unsuitable candidate.^8^ Hence, we began our investigations by assessing differently substituted phenanthrolines as ligands in a model reaction of our catalytic system (Figure 2A), where an alkyl iodide (**1**) transforms to the corresponding alcohol (**2**) through an analogous Ni–alkyl intermediate.^7b^ The catalytically active ligand **L1** afforded a 50% yield of **2**, under similar reaction conditions. Motivated by a recent study by Martin and co-workers on the carboxylation of organonickel complexes, we tested **L2**, anticipating that it would facilitate the isolation of putative Ni­(I)-alkyl species if successful;^9^ however, traces of product were obtained (<5%). At this point, we speculated that in addition to steric protection of the Ni center, the Mes group endowed **L2** with a certain rigidity, thus potentially minimizing reactivity. After a screening of ligands (see the Supporting Information for details), we synthesized the novel structure **L3**, which is characterized by having two biphenyl units in the 2,9 positions of the phenanthroline, thus inferring steric protection while introducing flexibility to the flanking groups. When it was tested in catalysis, a satisfactory 42% yield of **2** was obtained (Figure 2A), establishing it as the ligand of choice for further organometallic studies.

**Figure 2:**
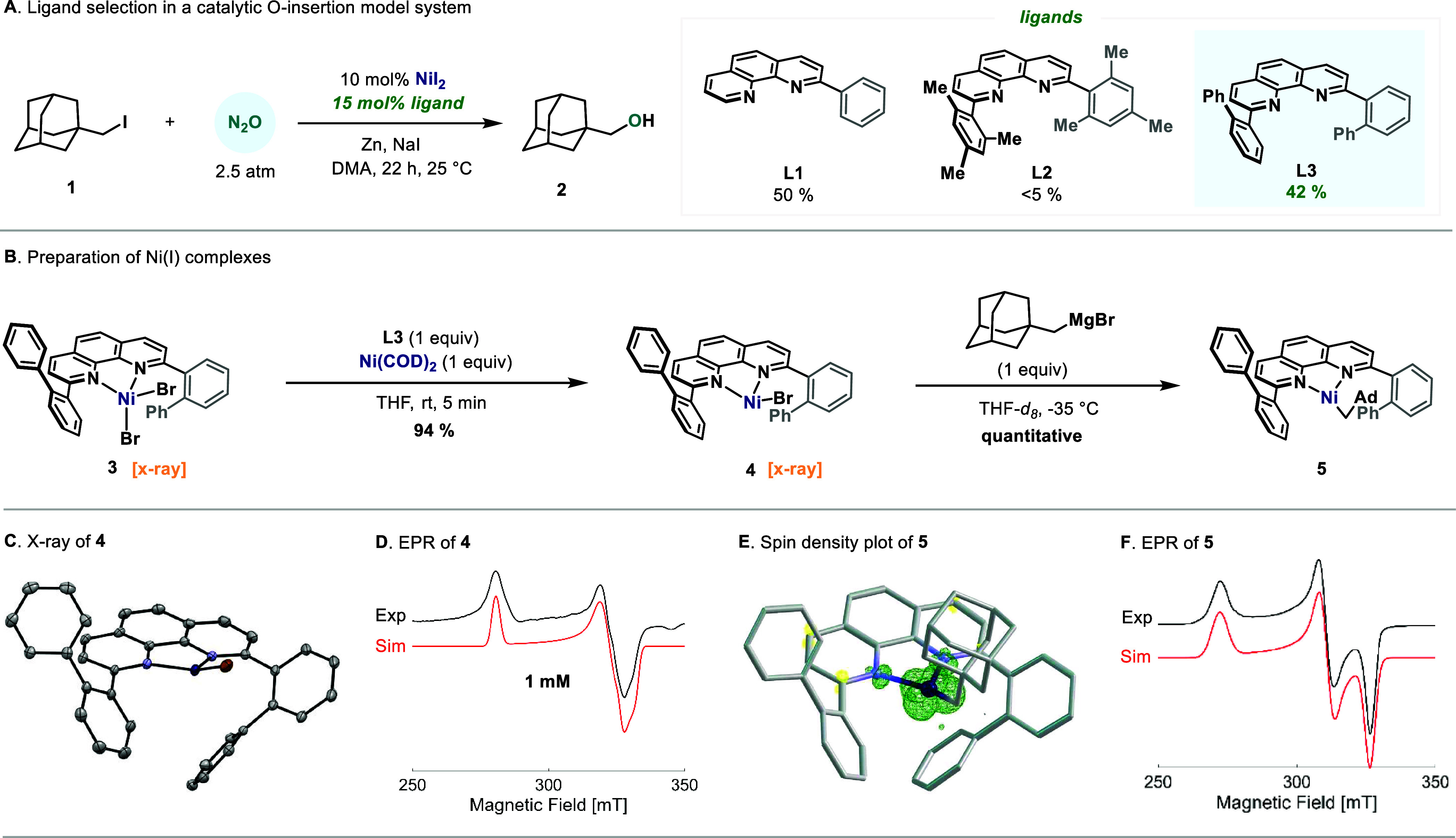
Catalytically relevant Ni­(I) species: ligand evaluation at the model reaction, synthesis, and spectroscopic characterization. Ad: 1-adamantyl.

Following Martin and co-workers’ approach,^9a^ we planned to access the parent **L3**–Ni^I^–C­(sp^3^) species via comproportionation of Ni­(II) and Ni(0) followed by a transmetalation with an alkyl Grignard. The great solubility of **L3** allowed facile complexation with Ni­(dme)­Br_2_ (dme = 1,2-dimethoxyethane), affording **3** as a THF-soluble pink powder (Figure 2B). The enhanced solubility also facilitated the growth of crystals suitable for SC-XRD (single crystal X-ray diffraction) analysis, which confirmed a monomeric, pseudotetrahedral nickel­(II) complex in the solid state (Figure S21). The cyclic voltammogram of **3** displayed a reversible redox event at −1.30 V vs Fc^+^/Fc, indicating an accessible Ni^II^/Ni^I^ potential (Figure S3).^10^ Indeed, reaction of **3** with Ni­(COD)_2_ in the presence of **L3** (1.0 equiv) in THF at 25 °C afforded **4** in almost quantitative yield (Figure 2B).^11^ Crystals of **4** suitable for SC-XRD ) were obtained, thus revealing the monomeric and trigonal geometry of the complex depicted in Figure 2C. Interestingly, the biphenyl groups spatially arrange in an *anti* configuration, protecting the Ni center from one side, while leaving open space for reagent approach at the other. In addition, the geometry imposed by the biphenyl groups force the Ni center out of the phenanthroline plane with a distance to the plane of 0.377 Å and the bromide out of the N,N,Ni-coordination plane by 0.707 Å. Compound **4** is paramagnetic and features broad signals by ^1^H NMR. The absence of **3**, even at 60 °C as judged by ^1^H NMR, confirms its redox stability toward disproportionation, unlike less hindered related bipyridine Ni­(I)–halide complexes (see the Supporting Information for details).^12^ Continuous wave (CW) EPR analysis at X-band (9.4 GHz) of a frozen sample of **4** in toluene revealed a nickel-centered radical (Figure 2D), consistent with similar tricoordinated Ni­(I) complexes reported.^8b,9a,13^ The slightly rhombic *g*-tensor [2.093(2) 2.142(2) 2.458(2)] exhibits the trend *g*_*z*_ > *g*_x,y_ > *g*_e_, indicating that the unpaired electron resides in the 3d_*x* ^2^–*y* ^2^_ orbital of the nickel. The broadening of the line width can be attributed to unresolved hyperfine couplings arising from the interaction with the Br ligand (nuclear spin *I* = 3/2, see the Supporting Information for details).^14^ Addition of the alkyl Grignard to **4** afforded a clean LNi­(I)–alkyl EPR spectrum, which we attributed to **5** (Figure 2E,F). Here, an increase of the rhombicity of the *g*-tensor [2.067(2) 2.171(3) 2.480(3)] was observed, in agreement with similar reported tricoordinated Ni­(I)–alkyl complexes.^8b,9a,13a^ A spin density plot revealed the position of the radical primarily at the d_*x* ^2^–*y* ^2^_ orbital of the nickel atom, akin to complex **4**. Spin integration against a CuSO_4_ external standard revealed the formation of **4** to proceed in quantitative yields. Analysis of the mixture by ^1^H NMR supported the absence of **3** or **4**. Control experiments revealed the absence of alcohol **2** upon the acidic quench of **5**.

With access to a well-defined, monomeric Ni­(I)–alkyl species armed with catalytically competent ligand **L3**, we assessed its reactivity toward N_2_O. When exposing a mixture of **5** in THF to 1.5 atm of N_2_O at 25 °C, N_2_ evolution was observed in less than 1 min, which was confirmed by GC-TCD of the headspace (Figure 3A). Further analysis of the reaction mixture also revealed complete consumption of **5**—as judged by EPR and ^1^H NMR— in favor of a new set of signals corresponding to complex **4** (Figure 3A).

**Figure 3:**
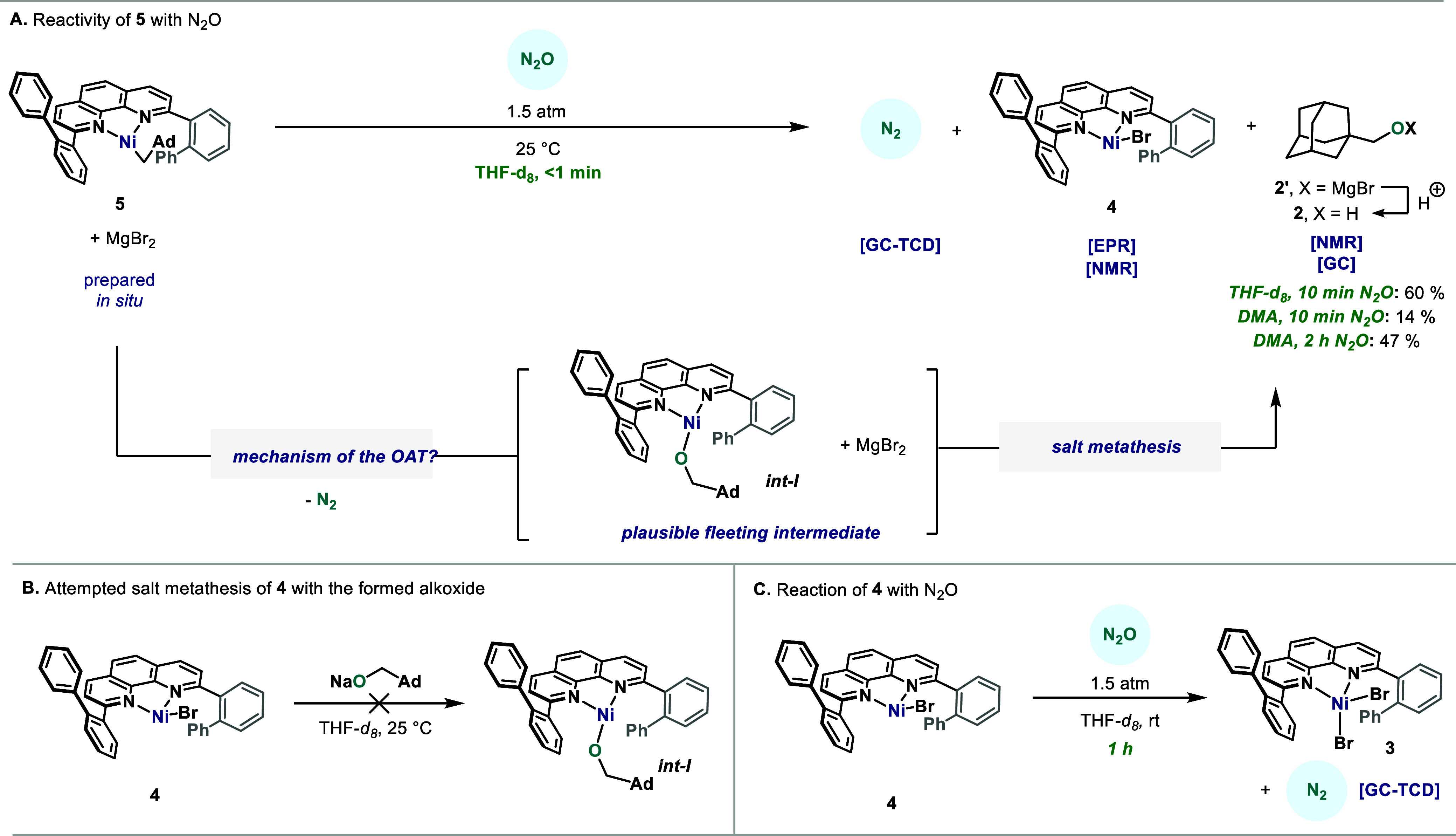
Reactivity of low-valent Ni-complexes with N_2_O.

After the mixture was stirred for 10 min under 1.5 atm of N_2_O, acidic quench under argon provided alkyl alcohol **2** in 60% yield (NMR and GC-FID). In light of this result, we hypothesized that **5** is indeed able to activate N_2_O and subsequently undergo C–O bond formation, leading to a putative **L3**–Ni^I^–(OR) species, which upon rapid salt metathesis with the MgBr_2_ present in the mixture delivers the observed **4**. Such a behavior is consistent with previously reported salt metathesis from related Ni­(I) carboxylates (Ni^I^–OCOR).^9a^ Indeed, attempts to synthesize ***int-I*** through salt metathesis between **4** and NaOCH_2_Ad were unsuccessful (Figure 3B, further details in the Supporting Information), leaving **4** unreacted. Although the thermal instability of **5** precluded its MgBr_2_-free isolation, the yield of alcohol **2** plummeted when the OAT experiment was performed in the presence of an additional equivalent of MgBr_2_ (see the Supporting Information for details), suggesting no indication of MgBr_2_ benefiting the C–O bond formation.

Interestingly, when **5** was prepared in DMA and subjected to a N_2_O atmosphere, the overall OAT was slower, forming only 14% of alcohol after 10 min. Importantly, when the mixture was allowed to react for 2 h, 47% **2** was obtained (Figure 3A). DMA is an enabling solvent for nickel catalysis,^15^ probably required to solubilize and stabilize **4**; yet, its coordinating abilities seem to hamper the approach of the weakly coordinating N_2_O to the Ni center thus slowing down the overall process.

We also confirmed that **4** reacts with N_2_O leading to compound **3** with concomitant formation of N_2_, albeit much slower (1 h, see the Supporting Information for details) than the reaction between **5** and N_2_O (<1 min).^16^ This points to a possible catalyst deactivation pathway, and hints to the required excess of reductant during catalysis.^7a,7b,17^

To assess the rapid formation of alcohols from complex **5**, we studied its reactivity using density functional theory^18^ with the r^2^SCAN-D4 method.^19^ The interaction between N_2_O and the Ni center of **5** resulted in three different binding modes for adduct ***add-I***: O-bound, N-bound, and side-on NN-bound with formation of a Ni–N–N three-center bond (Figure 4A; detailed descriptions in the Supporting Information). All modes are endergonic in the gas phase but become exergonic when the solvent (THF) is taken into account^20^ (Figure S26). This is due to the solvent model stabilizing the anisotropic charge distribution of intermediate ***add-I***, an absent effect in the gas phase. All binding modes are energetically close (Δ*G*_solv_ in the range of −6.1 to −2.2 kcal/mol) but are strongly differentiated in terms of electronic structure, because incipient oxidation of the Ni­(I) center and concomitant activation of N_2_O occurs *exclusively* in the case of O-bound N_2_O (Figure 4B and Table S11). This establishes a specific coordination requirement for subsequent reactivity to unfold. From the O-bound ***add-I***, loss of N_2_ leads to ***int-Ia*** that is thermodynamically more stable than the reactants (Δ*G*_solv_(**4**–**5**) = −27.0 kcal/mol) (Figure 4C). DFT calculations suggest that ***int-Ia*** is best described as a low-spin Ni­(II)–oxyl, with significant radical character on the O atom (see the Supporting Information for details). This intermediate is then primed for insertion of an O into the Ni–C­(sp^3^) bond. The corresponding transition state (**TS-I**) has a barrier of 7 kcal/mol for the concerted breaking of the Ni–C bond and insertion of O, leading to the strongly stabilized (by 41.6 kcal/mol) product ***int-I***.^21^ The subsequent salt metathesis with MgBr_2_ was computed to be thermodynamically favorable (Δ*G*_solv_ = –9.5 kcal/mol), implying that ***int-I*** might not be an observable intermediate. In summary, the reaction of **5** with N_2_O appears to proceed through a stepwise, low-valent OMVB-type mechanism. In our studies, there were no indications of (phen)^−^Ni^II^alkyl or (phen)^2–^Ni^II^alkyl intermediates.^22^

**Figure 4:**
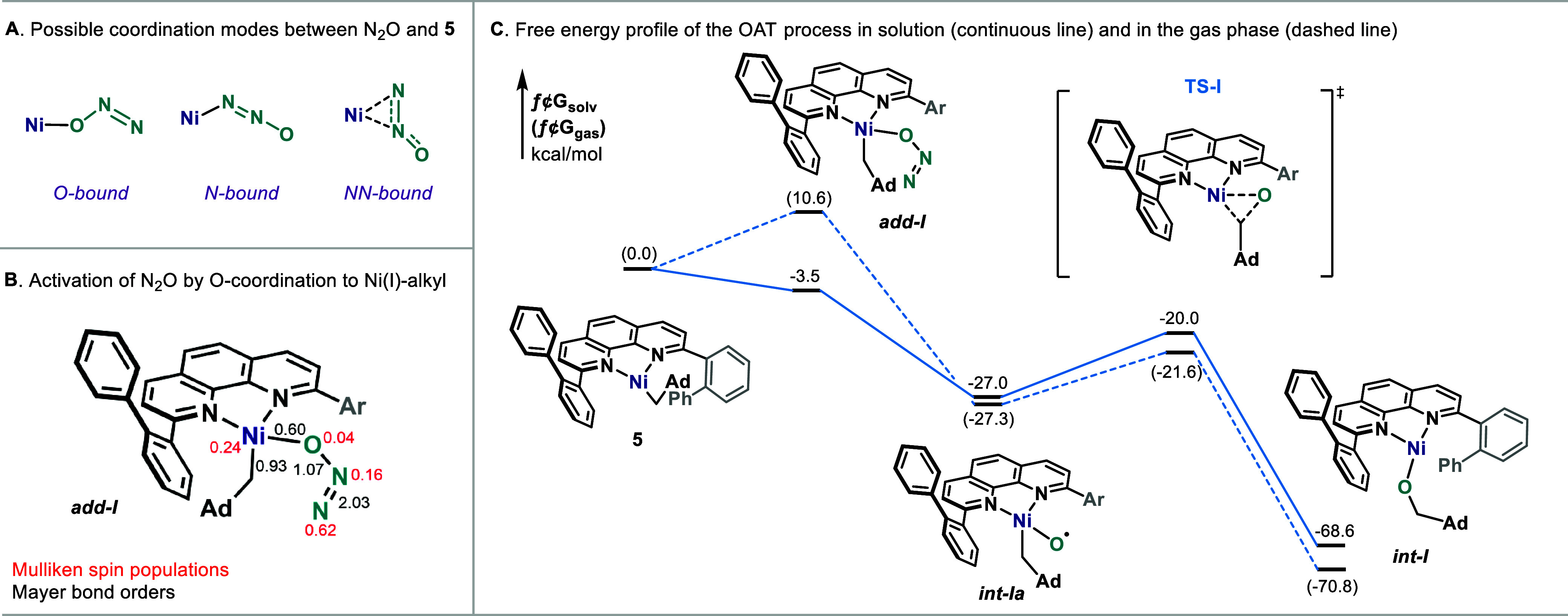
Theoretical analysis of the reaction of **5** with N_2_O. Ar: (1,1'-biphenyl)-2-yl.

In conclusion, we have successfully achieved the synthesis of a novel Ni­(I)–alkyl complex bearing a phenanthroline ligand (**5**), that enables a Ni-catalyzed alcohol synthesis with N_2_O as the O-source. Contrasting with the slow reactivity of less reducing Ni­(II)­dialkyl complexes toward N_2_O, we demonstrate that **5** rapidly reacts with N_2_O under ambient conditions leading to alcohol formation. Theoretical studies support a low-valent, stepwise OMBV mechanism via a low-spin Ni­(II)-oxyl intermediate.

While these results further support the involvement of Ni­(I)–alkyl species in catalysis, they also challenge the common assumption that prolonged reaction times stem from the inherently slow reactivity between N_2_O and the Ni center. Thus, low-valent Ni-catalysis holds promise for further development of fast protocols using N_2_O as O-source.

## Supplementary Material





## Notes

Experimental and characterization details, including ^1^H NMR and EPR spectra, crystallographic data and computational methods (PDF)
Cartesian coordinates of optimized structures (TXT)
