# Supplementary material for: Rapid Oxygen Atom Transfer at a Catalysis-Relevant Ni(I)–Alkyl Complex with N2O
Source: J Am Chem Soc. 2025 May 30;147(23):19438–43. doi: 10.1021/jacs.5c03351 (PMC12164269; doi:10.1021/jacs.5c03351)

Supporting Information

Rapid Oxygen Atom Transfer at Catalysis-relevant Ni(I)-alkyl Complex with N_2O

Ana Mateos-Calbet,[1] Paolo Cleto Bruzzese,[2] Markella Aliki Mermigki,[1] Alexander Schnegg,[2] Dimitrios A. Pantazis,[1]* and Josep Cornella[1]*

[1]Max-Planck-Institut für Kohlenforschung, Kaiser-Wilhelm-Platz 1, Mülheim an der Ruhr, 45470, Germany

[2]Max-Planck-Institut für Chemische Energiekonversion, Stiftstrasse 34–36, Mülheim an der Ruhr, 45470, Germany

*Corresponding authors: cornella@kofo.mpg.de, dimitrios.pantazis@kofo.mpg.de

Table of Contents

1. **General Information** 3
2. **Preparation of Ligands** 5
3. **Benchmark Reaction for Ligand Evaluation** 9
4. **Synthesis and Characterization Data of Nickel Complexes** 10
5. **Reaction of the *in situ* Formed Ni(Phen(BiPh)$_2$)CH$_2$Ad with N$_2$O** 17
6. **Effect of additional MgBr$_2$ on stoichiometric and catalytic experiments** 22
7. **Attempted Salt Metathesis of 4 with NaOCH$_2$Ad** 23
8. **Oxidation of 4 under N$_2$O** 26
9. **Crystallographic Data** 29
10. **DFT Study on the reaction of 5 with N$_2$O** 41
11. **References** 45
11. **NMR Spectra of new compounds** 47

1. General Information

Unless otherwise stated, all manipulations were performed under argon using standard Schlenk line techniques or in an MBraun argon-filled glove box.

Chemicals: N_2O was provided by Air Liquide containing less than 5 ppm N_2, 1 ppm H_2O and 1 ppm air and O_2. THF-*d*$_8$ was purchased from Eurisotop, degassed by repeated freeze-pump-thaw cycles, distilled from the proper drying agents, and stored over 4 Å molecular sieves. 4 Å molecular sieves were activated at 180 °C under high vacuum (1×10^{-3} bar) for 18 h. Anhydrous n-pentane, THF, Et_2O and toluene were distilled from appropriate drying agents and were transferred under argon. Anhydrous n-hexane and benzene (purchased from Millipore Sigma-Aldrich), as well as DMA for stoichiometric experiments (purchased from Thermo Scientific), were degassed by the freeze-pump-thaw method and stored over 4 Å molecular sieves. Unless otherwise noted, all reagents were obtained from commercial suppliers and used without further purification.

Instruments: Flash column chromatography was carried out on silica gel (VWR Silica gel 60, 40-63µm). NMR spectra were recorded using a Bruker AVIII HD 300 MHz, Bruker Advance III HD 400 MHz. ^1H and ^{13}C chemical shifts (δ) are given in ppm relative to tetramethylsilane (TMS), coupling constants (J) in Hz. The solvent signals were used as references and the chemical shifts converted to the TMS scale: for ^1H NMR: $CDCl_3$, δ 7.26 ppm; THF-*d*$_8$, δ 1.73 ppm; for ^{13}C NMR: $CDCl_3$, δ 77.16 ppm; THF-*d*$_8$, δ 25.37 ppm. ^1H NMR spectra of the paramagnetic complexes **4** and **5** were processed using a multipoint baseline correction with the segments algorithm in MestReNova. NMR yields were determined by using 1,3,5-trimethoxybenzene as internal standard. Multiplicities are described as s = singlet, br s = broad singlet, d = doublet, t = triplet, q = quartet, dd = doublet of doublets, td = triplet of doublets, m = multiplet. High-resolution mass spectra were obtained using Bruker APEX III FT-MS (ESI ionization source), Finnigan MAT 95 (EI ionization source), Thermo Scientific Q Exactive GC Orbitrap GC-MS/MS (GC-MS with EI ionization source), or Thermo Scientific Q Exactive Plus (APPI ionization source). Elemental analysis was conducted at Mikroanalytisches Labor Kolbe. GC-TCD measurements were performed on Agilent Technologies GC 7890B with a 30 m HP-Plot 5 Å Molsieves column. Electrochemical experiments were conducted using a Gamry Interface 1010E potentiostat. Continuous wave (CW) EPR measurements were performed at X-band (~9.4 GHz) using a MS-5000 (Magnettech GmbH, Freiberg Instruments) operating from 93.15 K up to room temperature (RT). The spectra were recorded with a typical magnetic field sweep range of 100 mT, field modulation of 0.5 mT, microwave power of 10 mW and a field modulation frequency of 100 kHz. Q-band (~34 GHz) CW EPR spectra were carried out with a Bruker Elexsys E580 EPR X-band spectrometer equipped with a homebuilt Q-band extension capable of delivering 34 GHz microwave radiation. Cryogenic temperatures were achieved using an Oxford CF935 Helium flow cryostat. For these cases, a sweep range of 400 mT, field modulation of 0.7 mT, microwave power of 2.0 mW and field modulation frequency of 100 kHz were employed. Spin quantifications were carried out using a regression model obtained from fitting the double integrated EPR signal of aqueous frozen solutions of $CuSO_4$ at known different concentrations. The concentrations were verified using the optical absorption band of the $CuSO_4$ in water occurring at 810 nm (extinction coefficient $\varepsilon = 12.3\ M^{-1}\ cm^{-1}$). [1] Double integrated EPR signals were scaled by the resonator Q-

factor difference as well as the different filling factor between the standards and the target samples. All spectra simulations were done using the Easyspin EPR simulation package. [2]

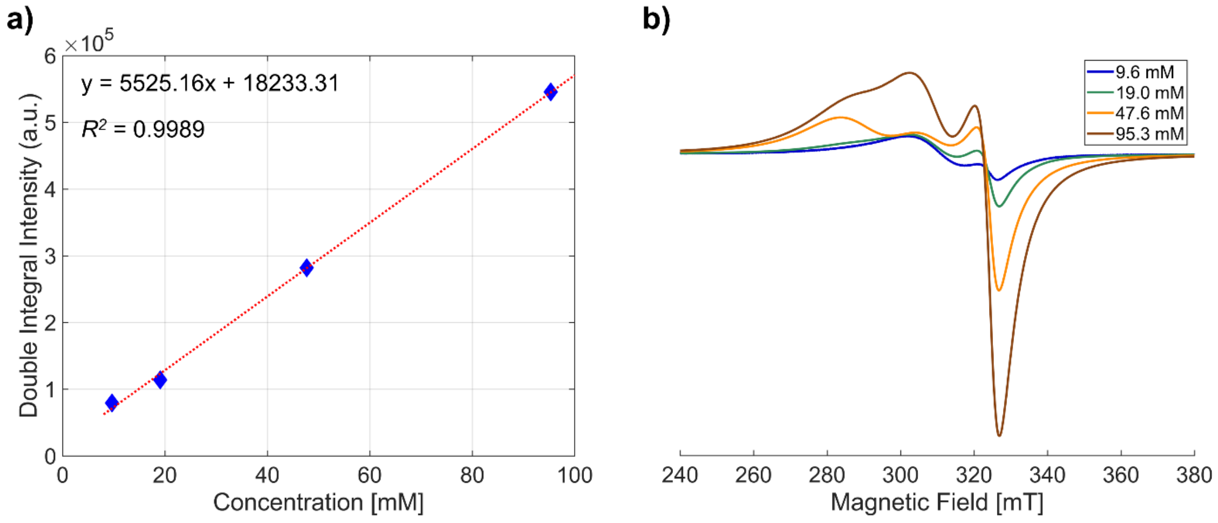

***Figure S1**: a) Standard curve for the double integrated intensity of $CuSO_4$ frozen solutions versus concentration. b) Corresponding EPR spectra of $CuSO_4$ frozen solutions measured at 100 K.*

Computational Details: For the DFT study, all calculations were performed with the Orca 5.0.3 software. [3] Optimizations were performed in gas phase using the r^2SCAN functional, [4] the def2-SVP basis set and def2-TZVP for the Ni center[5]. D4 was used for dispersion corrections. [6] Solvation corrections were made with the SMD continuum solvation scheme[7] for the THF solvent with single point calculations using the same level of theory. The Nudged-Elastic Band method in combination with the TS finding algorithm (NEB-TS) [8] was used for locating transition states.

Quantum-chemical calculations of relevant EPR parameters were carried out with Orca software package (5.0.4). [3] Starting from the structures optimized as described below, *g*-tensors were calculated in the frame of Density Functional Theory (DFT) using the double-hybrid B2PLYP functional[9] with the resolution of identity (RI) approximation. [10] The AutoAux keyword was used to build up auxiliary basis set. [11] A). The def2-TZVP basis set[5] was employed for C, Br and H atoms while the CP(PPP) [12] and EPR-III[13] basis sets were adopted for Ni and N atoms, respectively. The complete mean-field spin-orbit operator (SOMF) was used for treating the spin-orbit coupling (SOC). [14] Core electrons were not frozen in order to allow the computations of the analytical derivatives at MP2 level of theory.

2. Preparation of Ligands

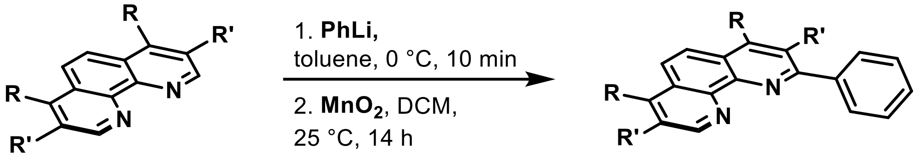

General Procedure A (GPA): The 2-phenyl substituted 1,10-phenanthroline ligands were prepared following a modified procedure for similarly substituted phenanthrolines. [15] The corresponding 1,10-phenanthroline derivative was previously dried under vacuum overnight and dissolved in toluene in a heatgun-dried Schlenk flask. Under vigorous stirring at 0 °C, phenyl lithium (PhLi, solution in ^{n}Bu$_2$O) was added dropwise until the color changed from light yellow to dark brown. From this point, one equivalent of PhLi was added dropwise. The mixture was stirred for 10 min allowing it to warm up to 25 °C, and water was added to quench the reaction. After extracting with CH_2Cl_2 (3 × 200 mL), the organic layers were combined and concentrated to 200 mL. MnO_2 was added in large excess and the suspension stirred at 25 °C for 14 h. The crude was purified *via* column chromatography.

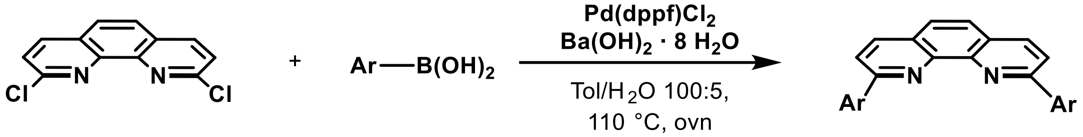

General Procedure B (GPB): The 2,9-aryl substituted 1,10-phenanthroline ligands were prepared following a reported procedure for similarly substituted phenanthrolines. [16] 2,9-Dichloro-1,10-phenanthroline, the aryl boronic acid, Pd(dppf)Cl_2 (dppf = 1,1′-Bis(diphenylphosphino)ferrocene), and $Ba(OH)_2 \cdot 8\ H_2O$ were introduced into a 250 mL pressure Schlenk flask, and toluene and water were added. The reaction mixture was sparged with N_2 for 30 min, and then stirred at 110 °C overnight. Upon cooling to ambient temperature, NH_4Cl (50 mL) and brine (10 mL) were added, and the mixture extracted with CH_2Cl_2 (3 × 100 mL). The organic layers were combined, dried over Na_2SO_4, filtered and evaporated. The crude was purified *via* column chromatography.

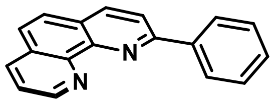

2-Phenyl-1,10-phenanthroline (L1)

Following the **GPA**, 1,10-phenanthroline (1.00 g, 5.55 mmol, 1 equiv.), toluene (200 mL), and PhLi (1.9 M in ^{n}Bu$_2$O, 2.92 mL, 5.55 mmol, 1 equiv.) were employed. Purification by silica pad filtration (first hexanes/EtOAc 1:1 to remove impurities, then EtOAc to flush product) yielded 2-phenyl-1,10-phenanthroline (**L1**, 1.10 g, 77 %) as a white powder.

^{1}H NMR (400 MHz, $CDCl_3$, 25 °C) δ 9.25 (dd, *J* = 4.3, 1.8 Hz, 1H), 8.37 – 8.29 (m, 3H), 8.26 (dd, *J* = 8.0, 1.8 Hz, 1H), 8.11 (d, *J* = 8.4 Hz, 1H), 7.80 (q, *J* = 8.8 Hz, 2H), 7.64 (dd, *J* = 8.0, 4.4 Hz, 1H), 7.58 – 7.52 (m, 2H), 7.50 – 7.44 (m, 1H).

^{13}C NMR (101 MHz, $CDCl_3$, 25 °C) δ 157.8, 150.6, 146.6, 146.3, 139.8, 137.0, 136.3, 129.5, 129.2, 128.9, 128.1, 127.7, 126.5, 126.4, 123.0, 120.8.

The spectroscopic data was in agreement with those reported in the literature. [17]

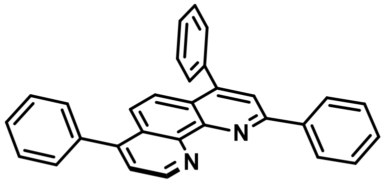

2,4,7-Triphenyl-1,10-phenanthroline (L4)

Following the **GPA**, bathophenantholine (2.00 g, 6.02 mmol, 1 equiv.), toluene (200 mL), and PhLi (1.8 M in ^{n}Bu$_2$O, 3.34 mL, 6.02 mmol, 1 equiv.) were employed. Purification by silica column chromatography (hexanes/EtOAc 7:3 to DCM with 2 % NEt_3) gave 2,4,7-triphenyl-1,10-phenanthroline (**L4**, 1.98 g, 80 %) as a white powder.

^{1}H NMR (400 MHz, $CDCl_3$, 25 °C) δ 9.29 (d, *J* = 4.5 Hz, 1H), 8.42 – 8.35 (m, 2H), 8.06 (s, 1H), 7.88 – 7.80 (m, 2H), 7.62 – 7.43 (m, 14H).

^{13}C NMR (101 MHz, $CDCl_3$, 25 °C) δ 157.13, 150.04, 149.34, 148.57, 147.20, 147.00, 139.89, 138.51, 138.25, 129.85, 129.83, 129.45, 128.92, 128.79, 128.73, 128.61, 128.58, 128.19, 126.95, 125.57, 124.07, 123.87, 123.47, 121.36.

HRMS (API-MS) calc. for $C_{30}H_{21}N_2$ $[M + H]^+$: 409.16992; found: 409.16990.

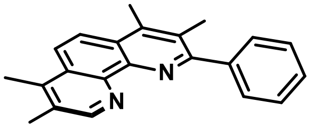

3,4,7,8-Tetramethyl-2-phenyl-1,10-phenanthroline (L5)

Following the **GPA**, 3,4,7,8-tetramethyl-1,10-phenanthroline (1.00 g, 4.23 mmol, 1 equiv.), toluene (150 mL), and PhLi (1.8 M in ^{n}Bu$_2$O, 2.35 mL, 4.23 mmol, 1 equiv.) were employed. Purification by silica pad filtration (first hexanes/EtOAc 7:3 to remove impurities, then EtOAc to flush product) yielded 3,4,7,8-tetramethyl-2-phenyl-1,10-phenanthroline (**L5**, 1.00 g, 76 %) as a white powder.

^{1}H NMR (400 MHz, $CDCl_3$, 25 °C) δ 8.93 (s, 1H), 8.09 – 8.02 (m, 2H), 7.67 – 7.60 (m, 2H), 7.46 (tt, *J* = 6.5, 1.1 Hz, 2H), 7.42 – 7.36 (m, 1H), 2.76 (s, 3H), 2.69 (s, 3H), 2.52 (s, 3H), 2.45 (s, 3H).

^{13}C NMR (101 MHz, $CDCl_3$, 25 °C) δ 160.2, 152.0, 145.3, 144.2, 142.5, 142.3, 141.4, 130.4, 129.8, 129.0, 128.1, 127.8, 127.2, 126.2, 122.2, 122.0, 17.9, 17.7, 15.3, 14.7.

HRMS (API-MS) calc. for $C_{22}H_{21}N_2$ $[M + H]^+$: 313.16992; found: 313.16964.

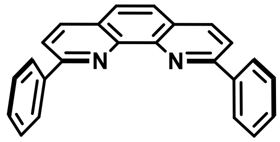

2,9-Diphenyl-1,10-phenanthroline (L6)

Following a modified procedure,[18] 2,9-dichloro-1,10-phenanthroline (1.50 g, 6.00 mmol, 1 equiv.), phenyl boronic acid (1.83 g, 15.0 mmol, 2.5 equiv.), Na_2CO_3 (11.1 g, 106 mmol, 18 equiv.), toluene (123 mL), water (53 mL) were added to a 500 mL two-neck round bottom flask equipped with a stir bar, a reflux condenser and an argon manifold and sparged with argon. Pd(dppf)Cl_2 (245 mg, 0.300 mmol, 5 mol%) was then added and the reaction then stirred under inert atmosphere at 80 °C for 20 h. After cooling to ambient temperature, the reaction mixture was extracted with CH_2Cl_2 (3 × 100 mL), the combined organic layers dried over $MgSO_4$, filtered, and evaporated. The resulting crude mixture was purified *via* silica gel column chromatography (hexanes/EtOAc 10:1 to 3:1) to give 2,9-diphenyl-1,10-phenanthroline (**L6**, 545 mg, 27 %) as a white solid.

^{1}H NMR ($CDCl_3$, 400 MHz, 25 °C): δ 8.46 (d, J = 7.2 Hz, 4H), 8.31 (d, J = 8.8 Hz, 2H), 8.14 (d, J = 8.4 Hz, 2H), 7.79 (s, 2H), 7.58 (t, J = 7.6 Hz, 4H), 7.48 (t, J = 7.4 Hz, 2H).

^{13}C NMR (101 MHz, $CDCl_3$, 25 °C) δ 156.9, 146.3, 139.6, 137.0, 129.5, 128.9, 128.0, 127.8, 126.1, 120.1.

The spectroscopic data was in agreement with those reported in the literature.[19]

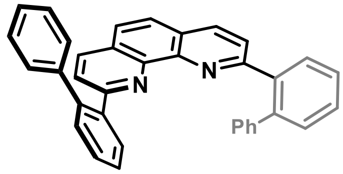

2,9-Di([1,1'-biphenyl]-2-yl)-1,10-phenanthroline (L3)

Following the **GPB**, 2,9-dichloro-1,10-phenanthroline (747 mg, 3.00 mmol, 1 equiv.), [1,1'-biphenyl]-2-ylboronic acid (1.78 g, 9.00 mmol, 3 equiv.), Pd(dppf)Cl_2 (329 mg, 0.450 mmol, 0.15 equiv), and $Ba(OH)_2 \cdot 8\ H_2O$ (4.73 g, 15.0 mmol, 5 equiv.), toluene (100 mL) and water (5 mL) were employed. Purification *via* silica column chromatography (2.5 % NEt_3, hexanes/EtOAc 95:2.5 to 9:1) gave **L3** (1.20 g, 83 %) as a white powder.

^{1}**H NMR** ($CDCl_3$, 400 MHz, 25 °C): δ 8.16 (dd, *J* = 7.6, 1.5 Hz, 2H), 7.87 (d, *J* = 8.4 Hz, 2H), 7.67 (s, 2H), 7.59 (td, *J* = 7.4, 1.6 Hz, 2H), 7.56 – 7.45 (m, 4H), 7.35 – 7.28 (m, 4H), 7.24 (dt, *J* = 4.7, 1.8 Hz, 6H), 7.17 (d, *J* = 8.3 Hz, 2H).

^{13}**C NMR** (101 MHz, $CDCl_3$, 25 °C) δ 159.5, 146.5, 141.8, 140.8, 140.2, 134.8, 132.3, 130.6, 130.2, 128.9, 128.3, 128.0, 127.2, 126.9, 126.2, 125.4.

HRMS (API-MS) calc. for $C_{36}H_{25}N_2$ $[M + H]^+$: 485.20122; found: 485.20113.

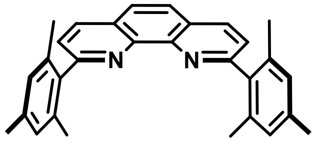

2,9-Dimesityl-1,10-phenanthroline (L2)

Following the **GPB**, 2,9-dichloro-1,10-phenanthroline (747 mg, 3.00 mmol, 1 equiv.), mesityl boronic acid (1.48 g, 9.00 mmol, 3 equiv.), Pd(dppf)Cl_2 (329 mg, 0.450 mmol, 0.15 equiv.), and $Ba(OH)_2 \cdot 8\ H_2O$ (4.73 g, 15 mmol, 5 equiv.), toluene (100 mL) and water (5 mL) were employed. Purification *via* silica column chromatography (5 % NEt_3, hexanes/EtOAc 80:15) gave 2,9-dimesityl-1,10-phenanthroline (**L2**, 0.75 g, 61 %) as an off-white powder.

^{1}**H NMR** (300 MHz, $CDCl_3$, 25 °C) δ 8.27 (d, *J* = 8.2 Hz, 2H), 7.85 (s, 2H), 7.57 (d, *J* = 8.2 Hz, 2H), 6.92 (bs, 4H), 2.32 (s, 6H), 2.15 (s, 12H).

^{13}**C NMR** (101 MHz, $CDCl_3$, 25 °C) δ 160.2, 146.4, 138.3, 137.5, 136.4, 135.8, 128.6, 127.2, 126.3, 125.2, 21.2, 20.8.

The spectroscopic data was in agreement with those reported in the literature. [16]

3. Benchmark Reaction for Ligand Evaluation

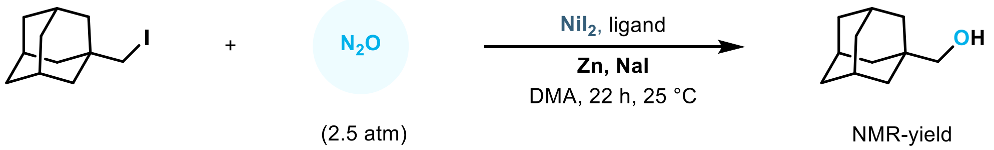

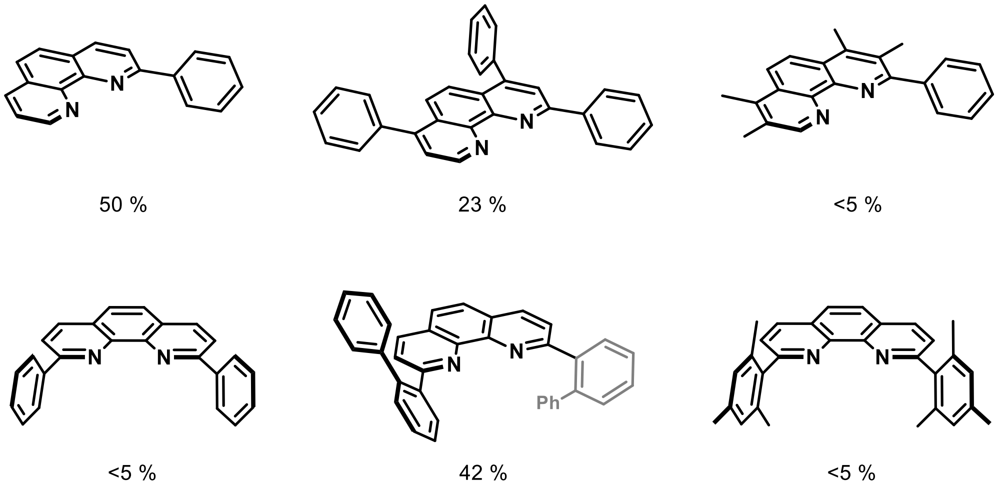

Figure S2: *Performance of differently substituted phenanthroline ligands in the Ni-catalyzed formation of an alkyl alcohol from an alkyl iodide.*

In an argon-filled glovebox, 1-(iodomethyl)adamantane (28 mg, 0.10 mmol, 1.0 equiv.), NiI_2 (3 mg, 1 µmol, 10 mol%), ligand (0.015 mmol, 15 mol%), Zn (26 mg, 0.40 mmol, 4 equiv.), and NaI (23 mg, 0.15 mmol, 1.5 equiv.) were introduced into a heatgun-dried pressure finger Schlenk flask. Using a three-way key, the tube was evacuated and refilled with N_2O. DMA (0.4 mL) was added while stirring under N_2O flow, then the flask was closed and the N_2O pressure increased to 2.5 atm. The mixture was stirred at ambient temperature for 22 h, and then quenched under argon with HCl in Et_2O (1 mL, 1 M). Water (2 mL) and 1,3,5-trimethoxy benzene (17 mg, 0.10 mmol, 0.33 equiv) as the internal standard were added, and the crude mixture extracted with Et_2O (3 × 3 mL). The combined organic layers were dried over Na_2SO_4, filtered, and evaporated under reduced pressure. The obtained crude mixtures were assessed *via* ^1H NMR and GC-MS.

NMR The reported ^1H NMR signal for AdC***H*₂**OH in $CDCl_3$ was used to determine the yield. [20]

4. Synthesis and Characterization Data of Nickel Complexes

Synthesis and Characterization of 3

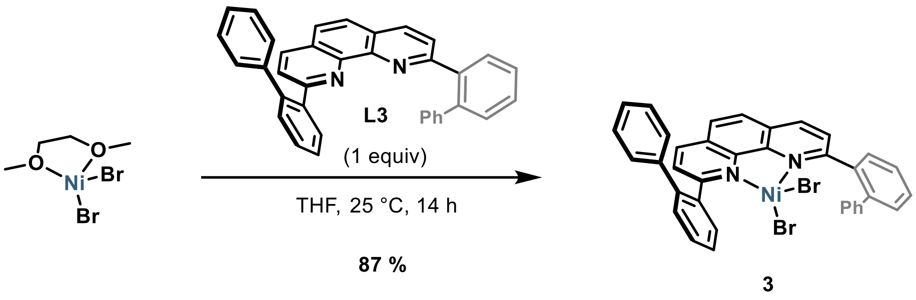

$NiBr_2$(glyme) (637 mg, 2.06 mmol, 1 equiv.), **L3** (1.00 g, 2.06 mmol, 1 equiv), and THF (50 mL) were added into a 250 mL Schlenk flask. The mixture was stirred for 30 min at 25 °C and sonicated for 14 h, giving a pink suspension. It was concentrated to a volume of 10 mL under vacuum. Et_2O (150 mL) was added, the suspension stirred for a *ca.*1 min, and the supernatant removed by decantation. The solid was washed with Et_2O (5 × 150 mL) and dried under vacuum to give **3** (1.26 g, 87 %) as a paramagnetic pink solid. Crystals suitable for single-crystal X-ray diffraction were grown from a $CDCl_3$ solution *via* benzene vapor diffusion.

^{1}H NMR (400 MHz, $CDCl_3$, 25 °C) δ 74.03, 46.95, 25.89, 22.94, 10.17, 8.06, 6.82, 6.15, 4.17, 3.27.

^{1}H NMR (400 MHz, THF-*d*$_8$, 25 °C) δ 73.24, 42.73, 25.20, 22.73, 9.74, 7.72, 6.86, 6.32, 4.77, 4.08.

Due to the paramagnetism of the sample, a ^{13}C NMR spectrum could not be measured.

HRMS (API-MS) calc. for $C_{36}H_{24}BrN_2Ni$ $[M - Br]^+$: 621.04708; found: 621.04779.

CV

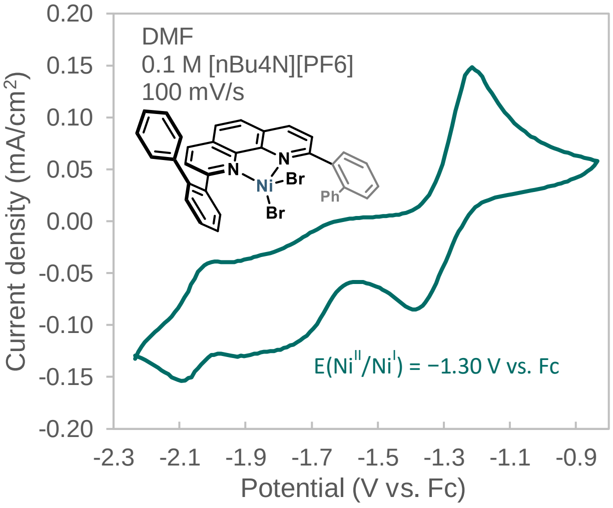

Figure S3: *Cyclic voltammogram of **3** in DMF (degassed and dried, 1 mM) at 25 °C. Set-up: glassy carbon working electrode (radius = 0.08 cm, geometric area = 0.02 cm^2), Pt wire counter electrode, Ag wire pseudo-reference electrode,*

[nBu$_4$N][PF$_6$] (0.1 M) as supporting electrolyte, 100 mV/s scan rate. Fc added as internal standard. E(Ni^{II}/Ni^{I}) = −1.30 V vs. Fc.

Synthesis and Characterization of 4

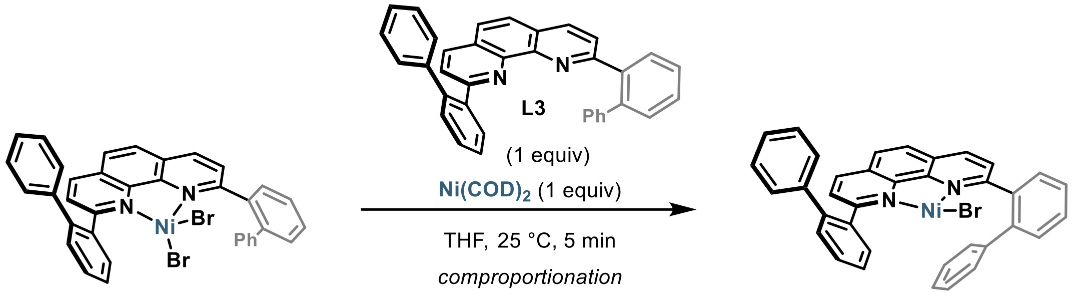

3 **94 %** **4**

In an argon-filled glovebox, **3** (500 mg, 0.711 mmol, 1 equiv.), **L3** (345 mg, 0.711 mmol, 1 equiv.), Ni(COD)$_2$ (196 mg, 0.711 mmol, 1 equiv.) and degassed THF (23 mL) were added into a heatgun-dried 50 mL Schlenk flask. The dark-blue mixture was stirred at 25 °C for 10 min. It was then filtered through a plug of dry Celite® into a heatgun-dried 100 mL Schlenk flask and solvents were removed under high vacuum. **4** was obtained as a paramagnetic dark blue powder (826 mg, 94 %). Crystals suitable single-crystal X-ray diffraction were grown *via* slow evaporation of the solvent from a THF solution.

^{1}H NMR (400 MHz, THF-*d*$_8$, 25 °C) δ 40.67 – 33.66 (bs), 33.50 – 25.03 (bs), 22.42 – 15.18 (bs), 11.77 (bs), 9.12 (bs), 8.29 (bs), 6.84 (bs), 6.29 (bs), 6.28 (bs), 4.82 (bs).

Due to the paramagnetism of the sample, a ^{13}C NMR spectrum could not be measured.

HRMS (API-MS) calc. for $C_{36}H_{24}BrN_2Ni$ [M]$^+$: 621.04708; found: 621.04744.

EPR *Note: a color change from dark blue to red was observed upon freezing. A similar behavior has been observed before for similar complexes.* [21]

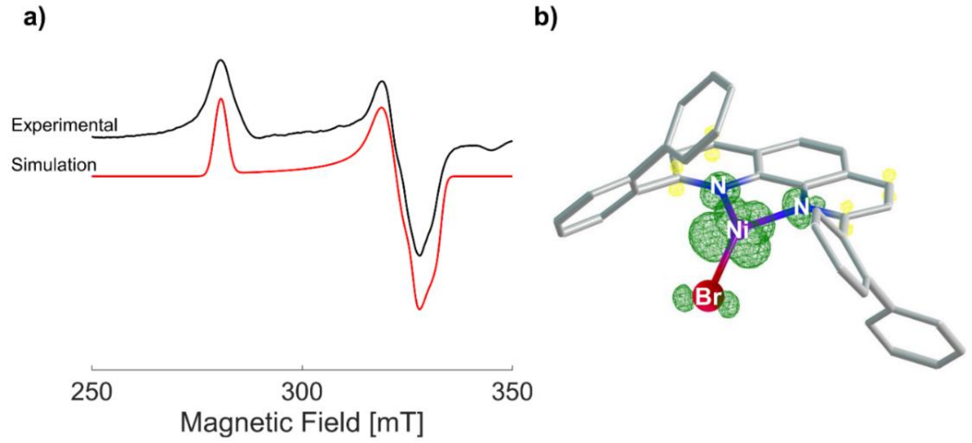

Figure S4*: a) Experimental and simulated X-band CW-EPR spectra of* ***4*** *(concentration 1 mM) measured in a frozen solution of toluene at 93 K. b). Computed spin-density plot for the optimized structure of* ***4*** *(contoured at ±0.002 electrons/a$_0$, positive spin density shown in green while negative one is shown in yellow).*

CW-EPR spectrum of **4** reported in Figure S4a exhibits a main signal with slightly rhombic feature characterized by $g_z > g_{x,y} > g_e$. [22] The pattern clearly indicates that the unpaired electron resides predominantly on the $3d_{x^2-y^2}$ orbital of the Ni^I (d^9 electron configuration, $S = 1/2$) center. Computer simulations of the spectrum reveals the contribution of a single species with main *g*-values, g_z = 2.458(2), g_y = 2.142(2) and g_x = 2.093(2), consistently with other paramagnetic Ni^I-halide complexes ligated with bidentate N-ligands[23, 24] with Y-shaped geometry. [25] The broadening of the linewidth of the spectrum is attributed to unresolved hyperfine couplings, mainly due to the interaction of the unpaired electron with the nuclear spin $I = 3/2$ of both bromine isotopes ^{79}Br and ^{81}Br (51% and 49% of natural abundance, respectively). Rough estimation of such interaction has been obtained by adding the contribution of one Br nucleus (in natural-abundance mixture) in the simulation procedure. A satisfactory fitting of the experimental broadening is achieved using the following hyperfine-tensor values A^{Br} = |[30(5) 20(5) 50(5)]| MHz (red trace in Figure S4a).

Although increasing the concentration of **4** in THF-*d₈* leads to a larger broadening of the EPR spectrum at X-band (Figure S5a), the contribution to the EPR signal identified at lower concentration in toluene still remains the dominant one. Inspection of the low-field region of the spectrum (75-160 mT) reveals the presence of the so-called half-field ($\Delta M_S = \pm 1$) transitions amenable to non-Kramer $S = 1$ species. Such signals, accounting for only 8 % of the total EPR spectrum can be tentatively assigned to a Ni^{II} species, arising from sample preparation.

Measurements at Q-band frequency allowed to disentangle minor contribution from at least other two Ni^I species (Ni^I(1) and Ni^I(2), violet and green trace in Figure S5b). The spin Hamiltonian parameters employed for the spectral simulations are summarized in Table S1. Inspection of the *g*-values shows that such minor Ni^I species are also characterized by a $3d_{x^2-y^2}$ singly occupied molecular orbital (SOMO).

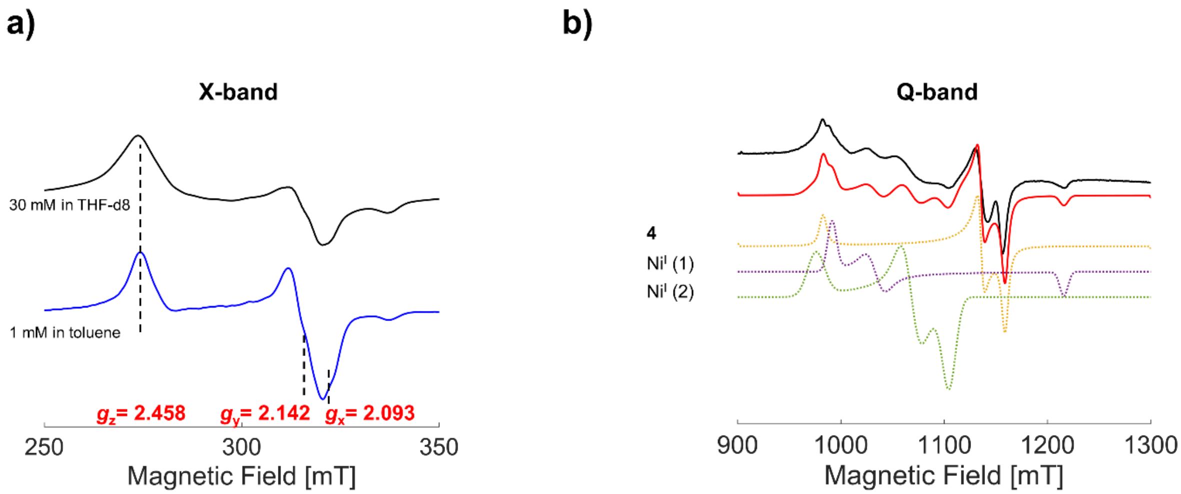

Figure S5: *a) Experimental X-band EPR spectra of **4** recorded at various concentration at 93 K. b) Experimental (black trace) and simulated (red trace) Q-band CW-EPR spectra of **4** (concentration 30 mM) measured at 70 K. The deconvolution of each contribution to the simulated pattern is shown with dashed lines.*

Table S1*: Experimental and computed spin Hamiltonian parameters for* ***4*** *compound at different concentrations. The hyperfine-tensor values are given in MHz.*

	Species	Weight	g_x	g_y	g_z		A_x	A_y	A_z
0.5 mM	***4***	100 %	2.093(2)	2.1425(3)	2.458(3)	$^{79,81}Br$	|33(5)|	|23(5)|	|47(2)|
30 mM	***4***	60 %	2.093(2)	2.1425(3)	2.458(3)	$^{79,81}Br$	|33(5)|	|23(5)|	|47(2)|
	Ni^{I} (1)	20 %	1.998(2)	2. 350(3)	2.450(2)	/	/	/	/
	Ni^{II} (2)	20 %	2.199(3)	2.275(3)	2.490(3)	/	/	/	/
DFT			2.118	2.167	2.383	$^{79,81}Br$	-13	-24	44

Stability assessment of 4

The stability of **4** in THF under argon was assessed by monitoring a 30 mM solution of **4** in THF-d_8 heated at 60 °C by EPR and NMR.

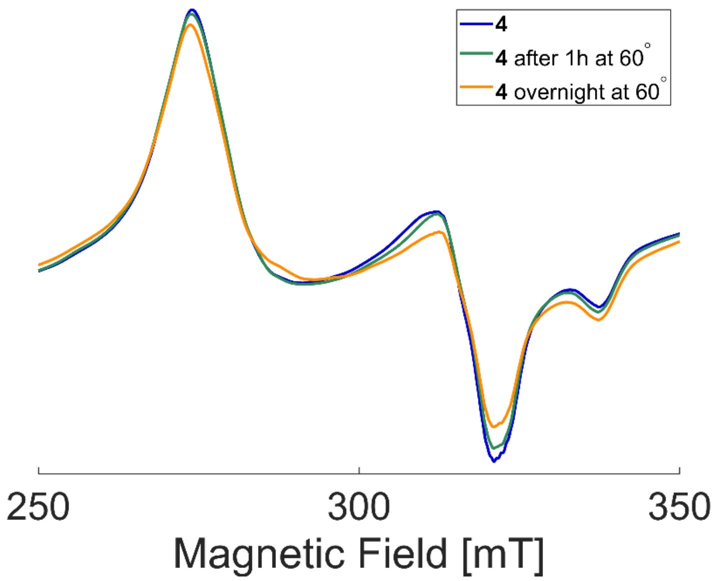

Figure S6*: Experimental X-band EPR spectra of* ***4*** *(~30 mM) recorded immediately after sample preparation (blue trace), after heating the sample for 1 h (green trace) or overnight (orange trace) at 333 K. Inspection of the relative EPR intensities shows that 80% of the signal is preserved even after overnight thermal treatment.*

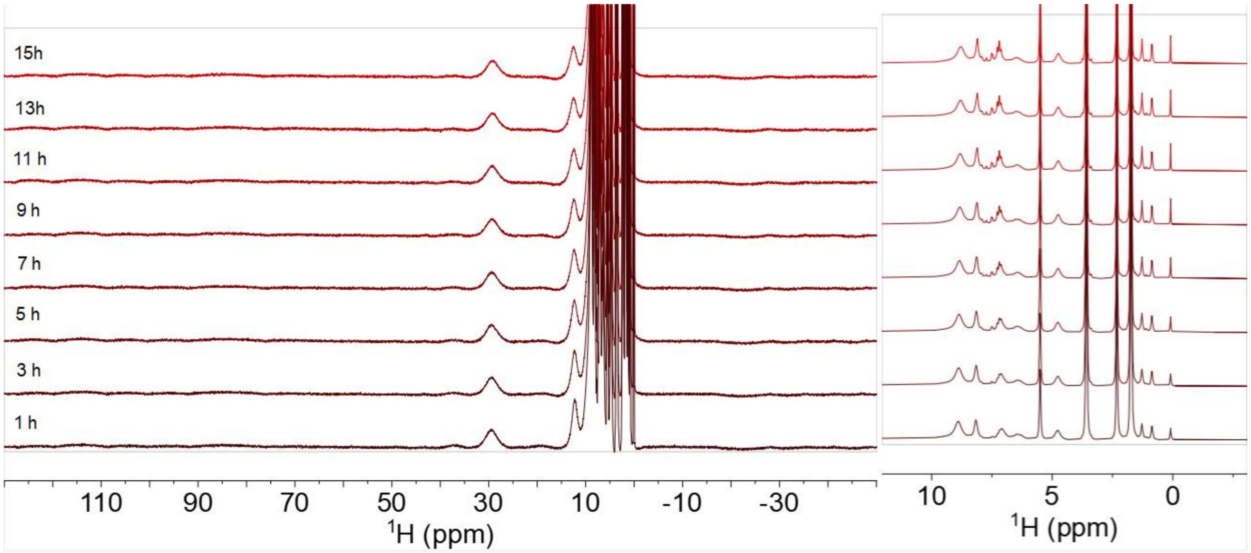

Figure S7: *^{1}H NMR spectra (400 MHz, THF-d_8, 30 mM, 25 °C) of a sample of **4** heated at 60 °C over time.*

No meaningful decomposition of **4** was observed in THF solution, even after heating at 60 °C overnight.

In situ Formation and Characterization of 5

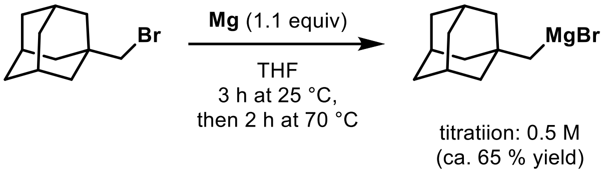

Preparation of the alkyl Grignard: Mg turnings were activated by adding three drops of concentrated HCl, washing them with water and acetone, and drying them under high vacuum overnight. The activated Mg (483 mg, 19.9 mmol, 1.1 equiv.), a sphere of I_2, and THF (0.5 mL) were added into a heatgun-dried three-neck 100 mL round bottom flask equipped with a reflux condenser and an argon manifold. In a separate 25 mL round bottom flask, 1-(bromomethyl)adamantane (4.50 g, 18.0 mmol, 1.0 equiv.) was dissolved in THF (17.5 mL). 2 mL of the prepared alkyl bromide solution were added dropwise to the three-neck round bottom flask and the mixture was shortly heated to 70 °C with a heatgun while stirring. Subsequently, the rest of the alkyl bromide solution was added. The mixture was heated to reflux (approx. 70 °C). After 2 h, the solution was allowed to cool to 25 °C and filtered into a flame-dried Schlenk flask *via* a cannula capped with filter paper. Titration of the obtained Grignard solution with 1,10-phenanthroline and menthol, as well as with I_2 in saturated LiCl-THF solution, revealed a Grignard concentration of 0.5 M. [26, 27] As a control experiment, a sample of the formed Grignard was quenched under argon with HCl in Et_2O (1 M). The resulting crude mixture was evaporated to dryness and analyzed *via* NMR and GC-MS: no oxygenated products could be detected.

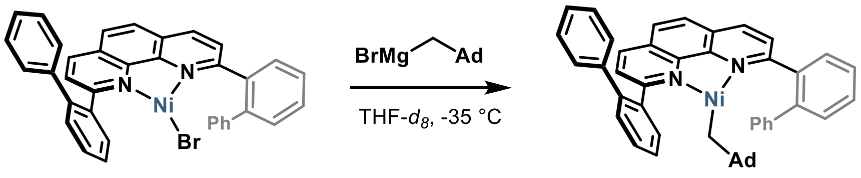

***In situ* preparation of the Ni(I)alkyl:** In an argon-filled glovebox, **4** (20 mg, 28 μmol, 1 equiv.) was dissolved in degassed THF-d_8 (1 mL) in a heatgun-dried Schlenk flask. The dark blue solution was cooled to −35 °C (acetone/dry ice bath), and the alkyl Grignard (0.5 M solution in THF, 0.11 mL, 0.057 mmol, 2 equiv.) was added dropwise. The mixture turned dark green, and was stirred at −35 °C for 5 min. For EPR and NMR analysis, a sample was transferred at this point into a heatgun-dried J-Young NMR tube filled with argon. As a control experiment, the *in situ* formed Ni(I)-alkyl was quenched under argon with HCl in Et_2O (1 M). The resulting crude mixture was then evaporated to dryness and analyzed *via* NMR and GC-MS: no oxygenated products could be detected.

If the Ni(I)alkyl sample was maintained at 25 °C, it gradually turned into a black suspension, an organic radical signal appeared in the EPR, and the NMR signals gradually diminished until they completely disappeared after ca. 20 min. These observations point to a decomposition *via* homolytic cleavage of the Ni-C bond. The thermal instability of **5** prevented its isolation. The loss of stability with more flexible substituted phenanthroline ligands was also observed by Martin *et al*.[24]

^{1}H NMR (400 MHz, THF-d_8, 25 °C) δ 49.90 – 41.00 (bs), 41.00 – 34.85 (bs), 11.82 (bs), 8.80 (bs), 8.45 (bs), 2.87 (bs), 1.43 (s).

Due to the paramagnetism of the sample, a ^{13}C NMR spectrum could not be measured.

HRMS could not be measured due to its low ionizability.

EPR

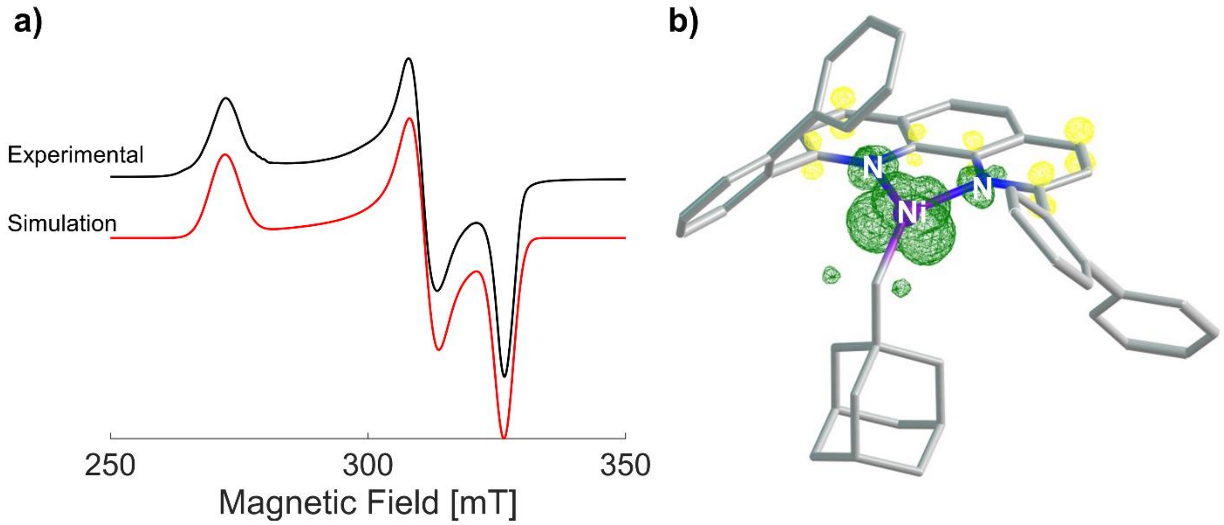

***Figure S8**: a) Experimental and simulated X-band CW-EPR spectra of **5** measured in a frozen solution of THF-d_8 at 93 K. b) Computed spin density plot for the optimized structure of **5** (contoured at ±0.002 electrons/a_0, positive spin density shown in green while negative one is shown in yellow). Ni-N-C bond angle is reported.*

The X-band CW-EPR spectrum of **5** reported in Figure S8a exhibits an increased rhombicity compared to the case **4** (see Figure S4 for comparison). Simulation of the spectrum provided the following *g*-values, g_z = 2.480(3), g_y = 2.171(3), g_x = 2.067(2), in good agreement with the previously reported Ni^{I}−alkyl complex synthesized by Somerville et al. [24] Calculation of the EPR parameters confirms the increased rhombicity of the **g**-tensor of the Ni^{I}-alkyl complex (**5**) with respect to the Ni^{I}-halide (**4**) one. The calculated **g**-tensor for the model of **5** exhibiting a Y-shaped geometry is in better agreement with the EPR findings compared to the T-shaped conformer (see Table S2). Noteworthy, the computed energy difference between Y-shaped and T-shaped conformers is only about 1 kcal/mol indicating that the conversion between them is almost barrierless. Double integration of the EPR signal, followed by spin quantification using the $CuSO_4$ standard curve, reveals that formation of **5** is quantitative. (32(3) mM of **5** are quantified by EPR from 30 mM of **4**).

***Table S2**: Experimental and computed spin Hamiltonian parameters for **5**.*

		g_x	g_y	g_z
**5**		2.067(2)	2.171(3)	2.480(3)
DFT	Y-shape (N-Ni-C angle = 133°)	2.090	2.235	2.421
	T-shape (N-Ni-C angle = 140°)	2.149	2.320	2.389

5. Reaction of the *in situ* Formed Ni(Phen(BiPh)$_2$)CH$_2$Ad with N$_2$O

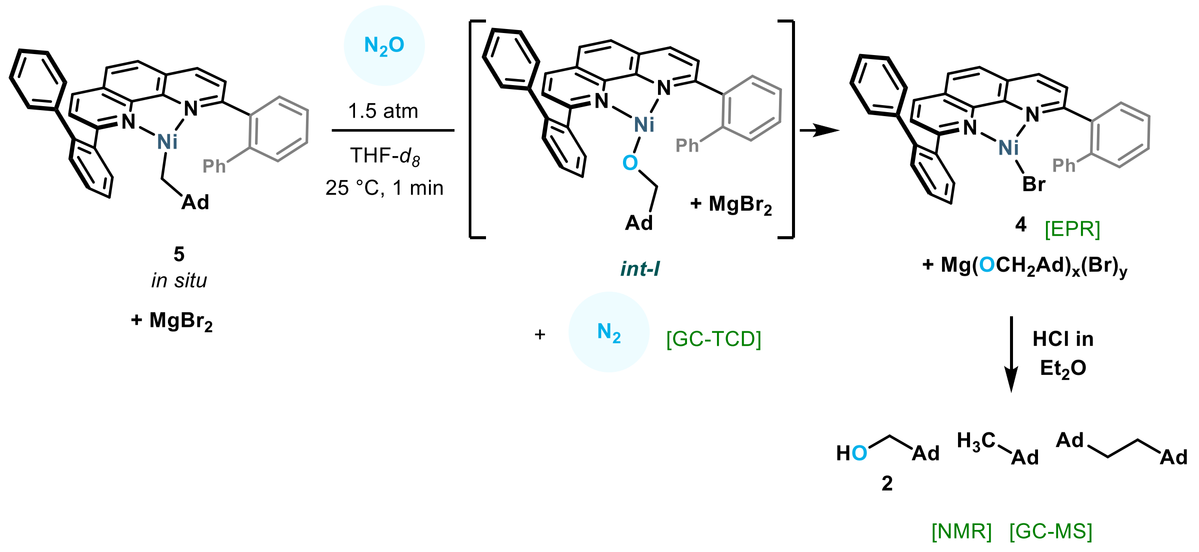

For the NMR and EPR analyses, the Ni(I)alkyl complex **5** was prepared *in situ* as described above, and a sample was transferred into a heatgun-dried J-Young NMR tube filled with argon. An EPR measurement was conducted to confirm the quality of the obtained Ni(I)alkyl. Subsequently, the sample in the tube was frozen in liquid nitrogen, and the headspace of the tube evacuated. The J-Young NMR tube was then closed, and the sample was thawed. Using a three-way key, the tube was refilled with N_2O (1.5 atm), and the tube was closed and inverted once. For EPR analysis, the sample was refrozen in liquid nitrogen after 1 min and measured. For NMR analysis, a ^1H NMR spectrum of the sample was recorded every 10 min for 2.5 h, with the first measurement having a delay of approx. 10 min after addition of N_2O.

EPR

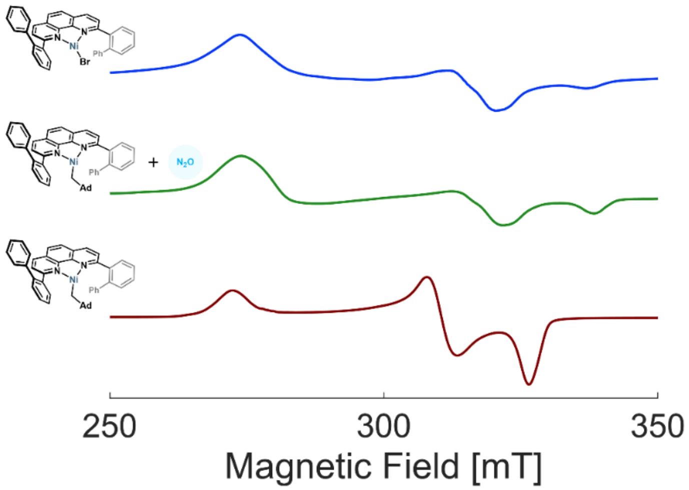

Figure S9: *Comparison of experimental X-band CW-EPR spectrum of **4** (blue trace), **5** after 1 min under N_2O (green trace) and **5** (maroon trace), all measured in a frozen solution of THF-d_8 at 93 K.*

Double integration of the green trace in Figure S9 reveals a yield of 43% for the formation of **4** after reaction of **5** with N_2O. However, since **4** oxidizes over time under these conditions (*vide infra*), this value cannot reflect the actual total amount of **4** formed in the course of this reaction.

NMR

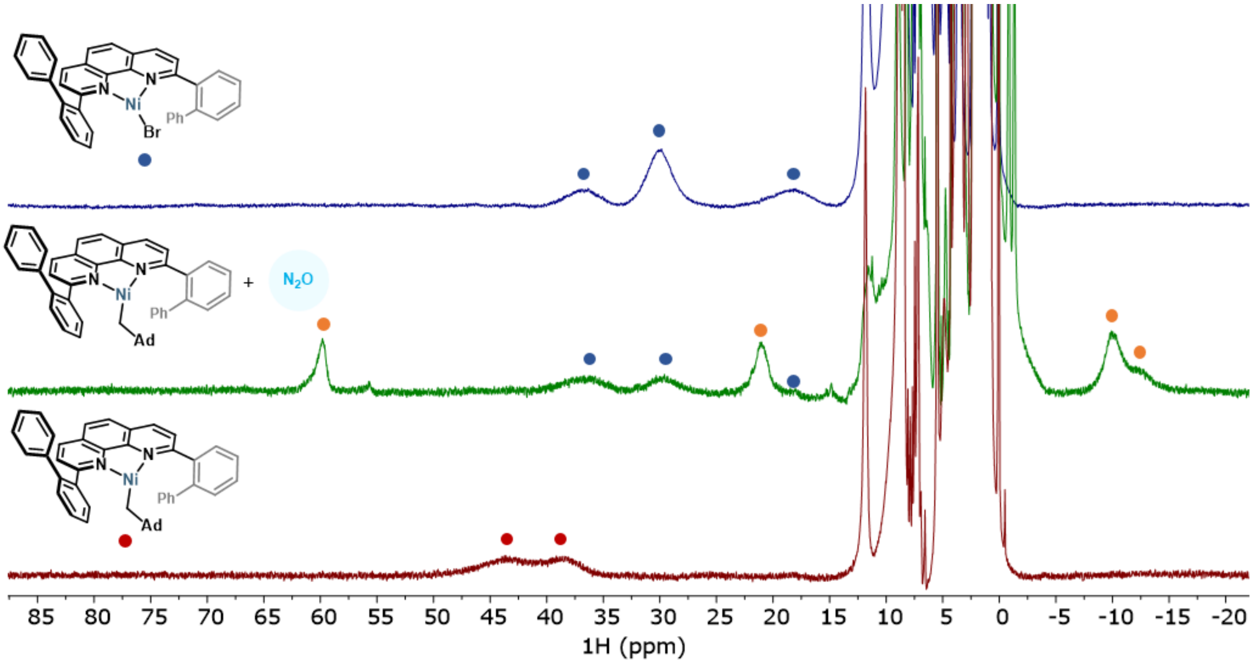

Figure S10: *¹H NMR (400 MHz, THF-d₈, 25 °C) of the reaction after 10 min under 1.5 atm N_2O (green trace). ¹H NMR of **5** (maroon trace) and of **4** (blue trace) included for comparison. Emerging unidentified species marked in orange.*

After 10 min at 25 °C under N_2O (1.5 atm), the Ni(I)alkyl signals (marked in red) are no longer discernible. Instead, signals at shifts observed for **4** (marked in blue) appear. Signals from an unidentified species appear as well (marked in orange), probably from an intermediate in the reaction.

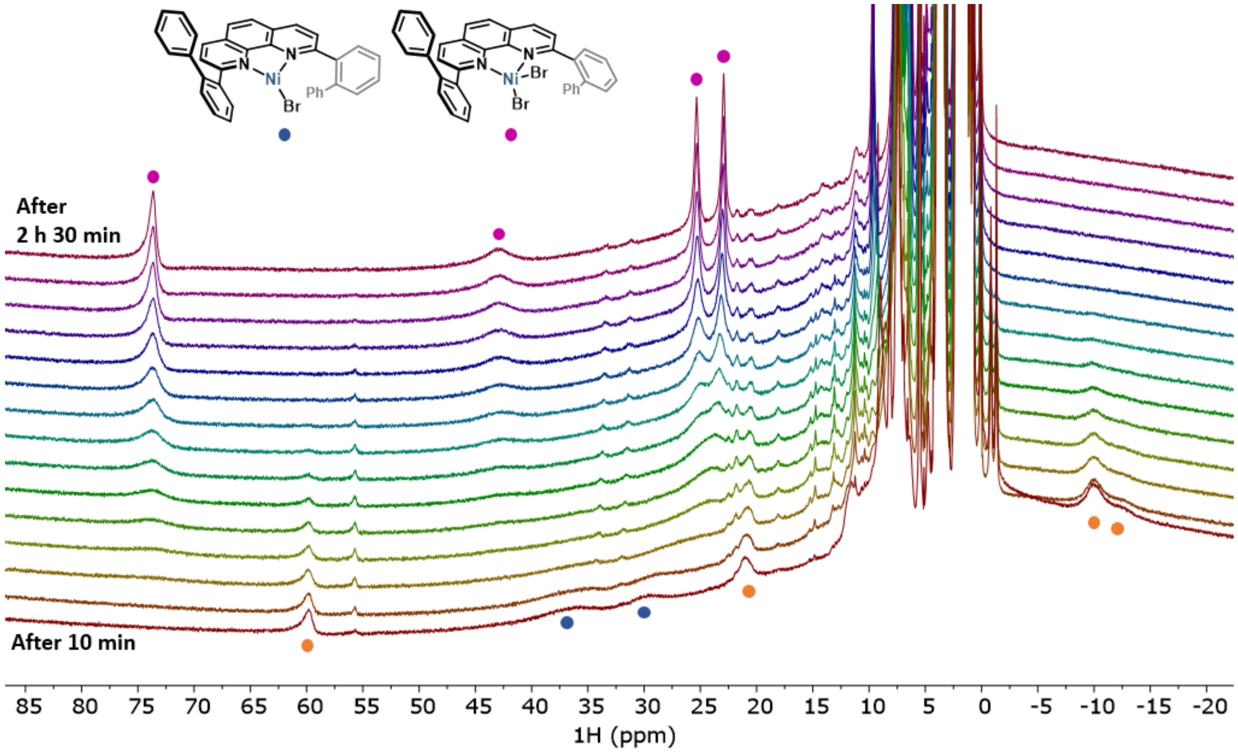

Figure S11: *^{1}H NMR (400 MHz, THF-d$_8$, 25 °C) measured every 10 min during the reaction under 1.5 atm N_2O.*

In the course of the next 2.5 h, **4** (marked in blue) and the intermediate species (marked in orange) disappear, while **3** (marked in magenta) is formed. This agrees with the slower, subsequent oxidation of **4** to **3** under N_2O (*vide infra*). **For the GC-TCD analysis of the reaction's headspace**, Ni(I)alkyl **5** was prepared *in situ* as described above but on a 0.057 mmol scale in a Schlenk flask equipped with a septum, and the resulting mixture frozen in liquid nitrogen. The headspace was evacuated, and after re-closing the Schlenk flask, the mixture was allowed to thaw. Using a three-way key, the Schlenk flask was refilled with N_2O (1.5 atm), and it was closed again. While stirring at ambient temperature, *gas evolution was observed within the first minute*. After 40 min, a sample of the headspace's gases was taken with a needle through the septum and analyzed *via* GC-TCD.

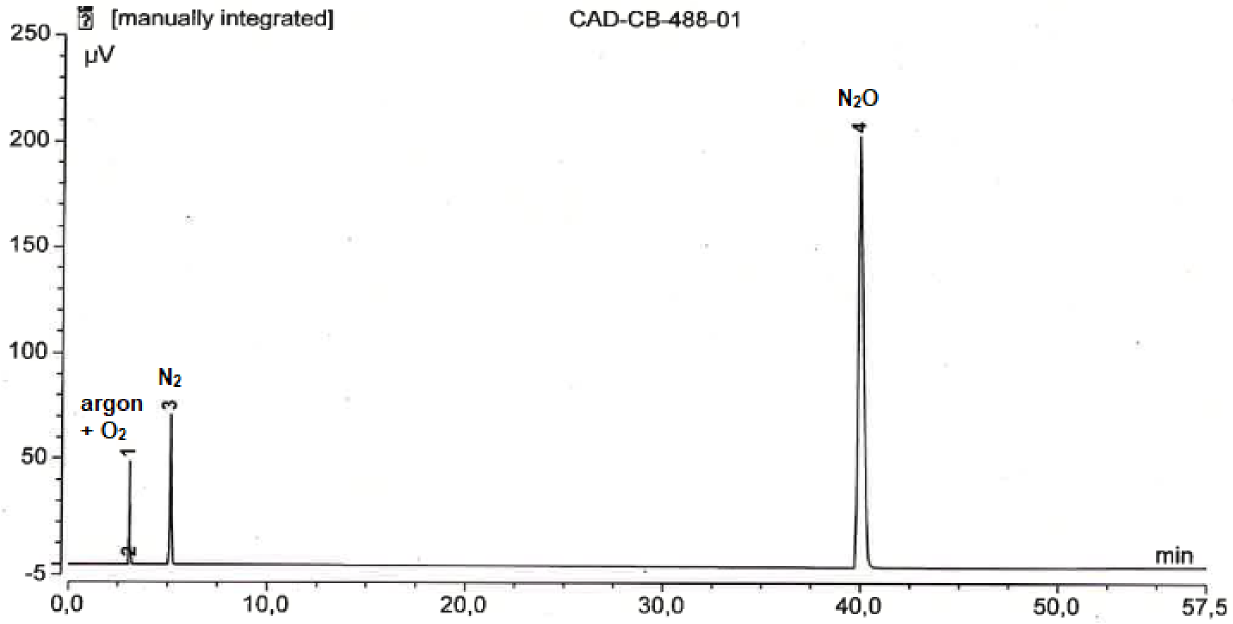

Figure S12: *GC-TCD chromatogram of the headspace of the reaction of* ***5*** *with N_2O after 40 min. Column: 27.7 m, HP-Plot 5 Å Molsieve 0.32/10.0 df G/764. Temperature: 220/30; 10 min iso 6/min 250 12/min 320, 5 min iso/ 250. Gas: 0.50 bar, He. Sample size: 250.0 μL.*

Table S3: *Peak data of the GC-TCD chromatogram of the headspace of the reaction of* ***5*** *with N_2O after 40 min.*

Peak	Retention time [min]	Area-%	Compound
1	3.07	2.83	Argon (no baseline separation)
2	3.13	0.11	O_2 (no baseline separation)
3	5.11	8.39	N_2
4	39.84	88.68	N_2O

For the quench experiment, Ni(I)alkyl **5** was prepared *in situ* as described above but in a Schlenk flask in the presence of 1,3,5-trimethoxybenzene (5 mg, 0.03 mmol, 1 equiv) as internal standard and adding one equivalent of alkyl Grignard (0.5 M in THF, 57 μL, 0.028 mmol), and the resulting mixture frozen in liquid nitrogen. The headspace was evacuated, and after re-closing the Schlenk flask, the mixture was allowed to thaw. Using a three-way key, the Schlenk flask was refilled with N_2O (1.5 atm), and closed again. Stirring at room temperature, *gas evolution was observed within the first minute*. After 10 min, the supplying gas was changed to argon, and HCl in Et_2O (1 M, 1 mL) was added under an argon flow. The resulting crude mixture was evaporated under high vacuum, re-dissolved in $CDCl_3$, and analyzed *via* ^1H NMR and GC-MS analysis. Alcohol **2** was formed in 60 % NMR yield.

NMR The reported ^1H NMR signal of AdC$\mathbf{H_2}$OH in $CDCl_3$ was used to determine the yield. [20]

GC-EI/MS

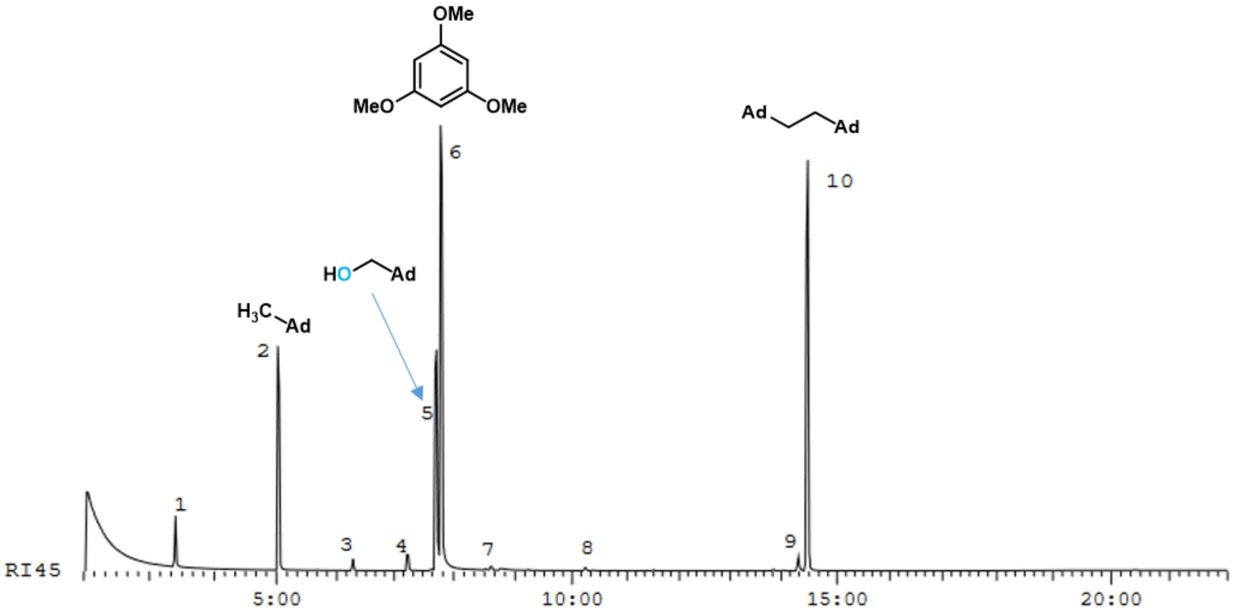

Figure S13: *GC-EI/MS chromatogram of the quenching experiment. Spectrometer: Q Exactive GC Orbitrap. Column: G211 XTI-5. Length: 30 m. Solvent: chloroform. Temperature programm: from 35 °C – 15 °C/min – to 285 °C – 5 min.*

Quenching of the employed Grignard in the presence of trimethoxybenzene as internal standard indicated that the detected dimer stems from the Grignard solution, probably being formed in during synthesis from Wurtz-like coupling side reactivity.

The experiment testing the reactivity of Ni(I)alkyl **5** with N_2O was repeated using dry and degassed DMA as solvent. After stirring the *in situ* formed Ni(I)alkyl in DMA under N_2O for 10 min and quenching with HCl in Et_2O, alcohol **2** was observed in 14 % yield. Stirring the *in situ* formed Ni(I)alkyl in DMA under N_2O for 2 h afforded 47 % of alcohol **2**.

6. Effect of additional $MgBr_2$ on stoichiometric and catalytic experiments

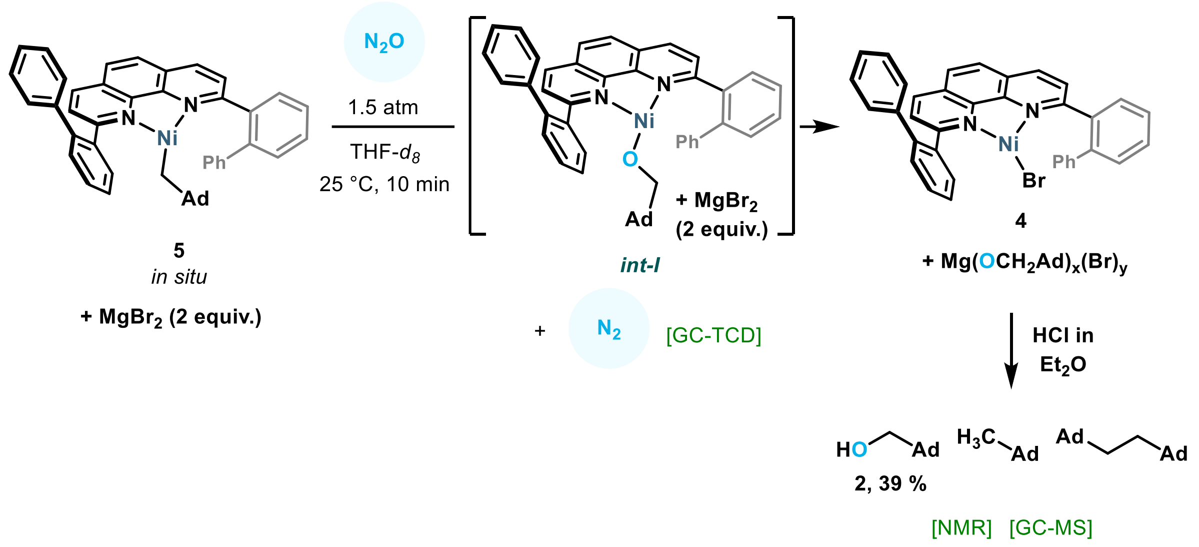

In an effort to assess whether the present $MgBr_2$ facilitates the observed OAT reaction, the quantification experiment (*vide supra*) was repeated in the presence of 1.0 additional equiv. of $MgBr_2$. The alcohol was detected in diminished yield relative to the model reaction without added $MgBr_2$, suggesting that the $MgBr_2$ does not have a positive effect on this reaction.

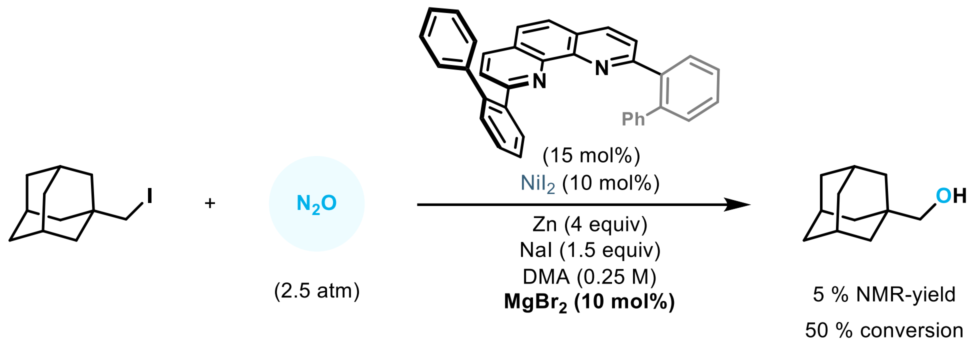

The effect of $MgBr_2$ was also investigated for the catalysis experiment, which was also repeated in the presence of equimolar amounts of $MgBr_2$ respective to Ni. In this case the alcohol yield dropped to 5%. Since the conversion was much lower than without $MgBr_2$, this Lewis acid seems to have a deleterious effect on other steps of the catalysis as well.

These results show no signs of $MgBr_2$ benefiting the N_2O activation and C–O bond formation.

7. Attempted Salt Metathesis of 4 with $NaOCH_2Ad$

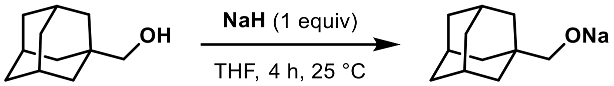

Preparation of $NaOCH_2Ad$: In a heatgun-dried 100 mL Schlenk flask equipped with an argon manifold, $HOCH_2Ad$ (1 g, 6 mmol, 1 equiv) and NaH (144 mg, 6.02 mmol, 1 equiv) were stirred in THF (20 mL) at ambient temperature. After 4 h, the volatiles were removed under high vacuum and the residue triturated with pentane and dried under high vacuum. The obtained alkoxide was used without further purification.

To determine the purity and yield of the crude product, 50 mg of the product were dissolved in THF-d_8 (2 mL) in a heatgun-dried Schlenk flask, MeI (19 µL, 0.30 mmol, 1 equiv) was added to the solution and the mixture stirred for 4 h at ambient temperature. ^{1}H NMR revealed a full conversion of the alkoxide to the methyl ether and 15 % residual MeI. This indicates a full deprotonation of the original alcohol and an 85 % yield of $NaOCH_2Ad$.

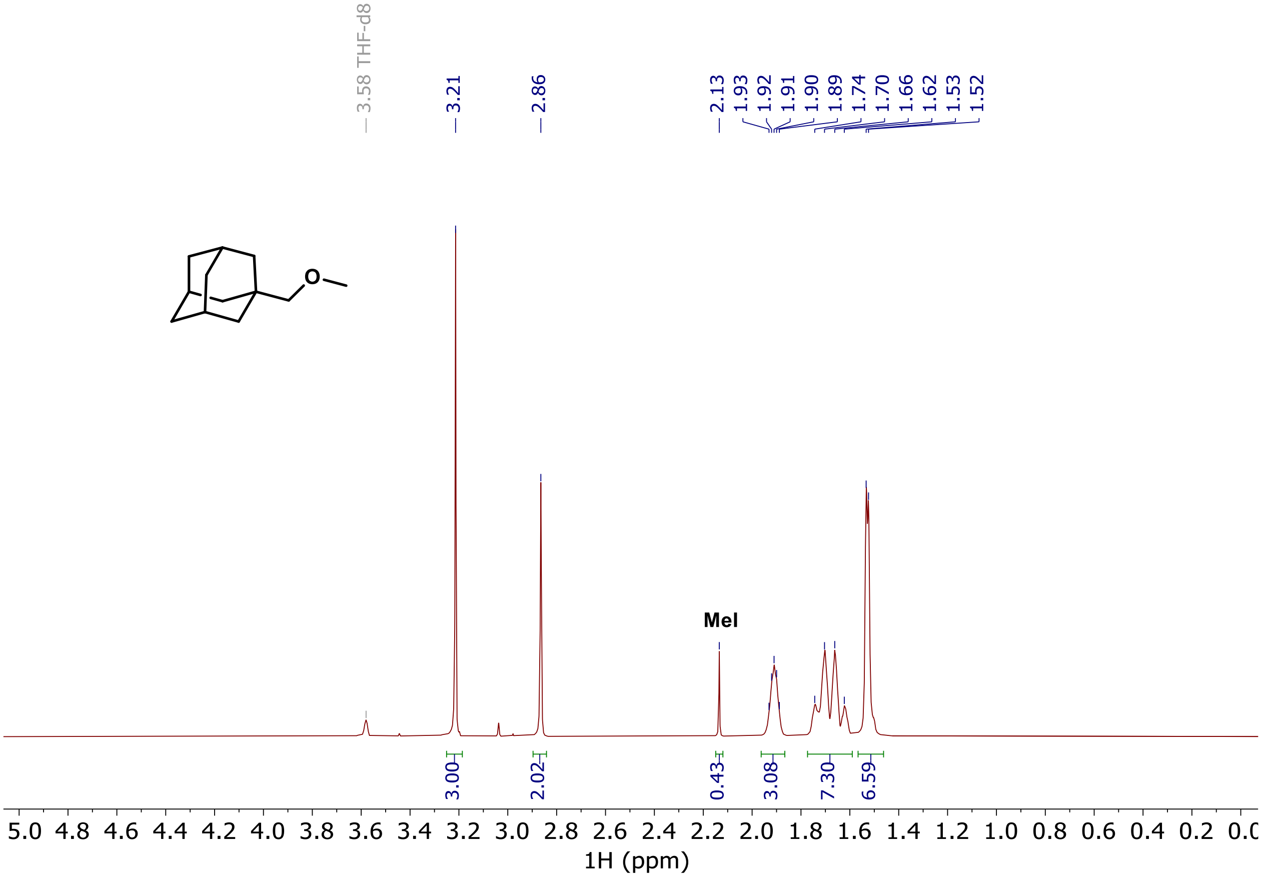

Figure S14: *^{1}H NMR (300 MHz, THF-d_8, 25 °C) of the mixture of $NaOCH_2Ad$ and MeI after 4 h.*

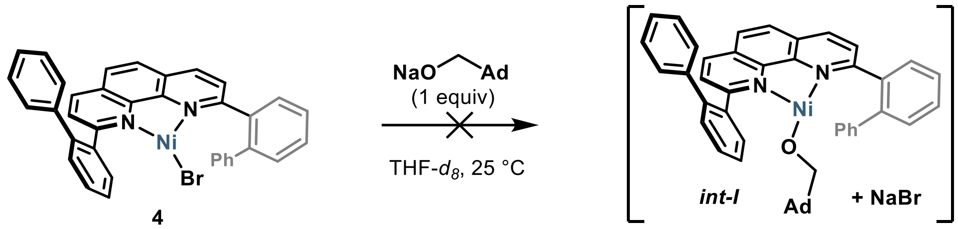

Attempted metathesis experiment: In an argon-filled glovebox, **4** (20 mg, 0.028 mmol, 1 equiv.) and $NaOCH_2Ad$ (85 %, 7 mg, 0.03 mmol, 1 equiv.) were dissolved in degassed THF-d_8 (1 mL) in an oven-dried glass vial and stirred at ambient temperature for 10 min. The resulting mixture was filtered through an HPLC filter into a heatgun-dried J-Young NMR tube filled with argon and analyzed *via* ^{1}H NMR and EPR. The tube was then heated to 60 °C for a period of 15 h, during which the sample was monitored by ^{1}H NMR. After that time, the tube was analyzed again by EPR.

Analysis of the organic products after acidic quench of the same experiment after 1 h heating at 60 °C did not detect any other species (e.g. the analogous aldehyde) besides alcohol **2**.

EPR

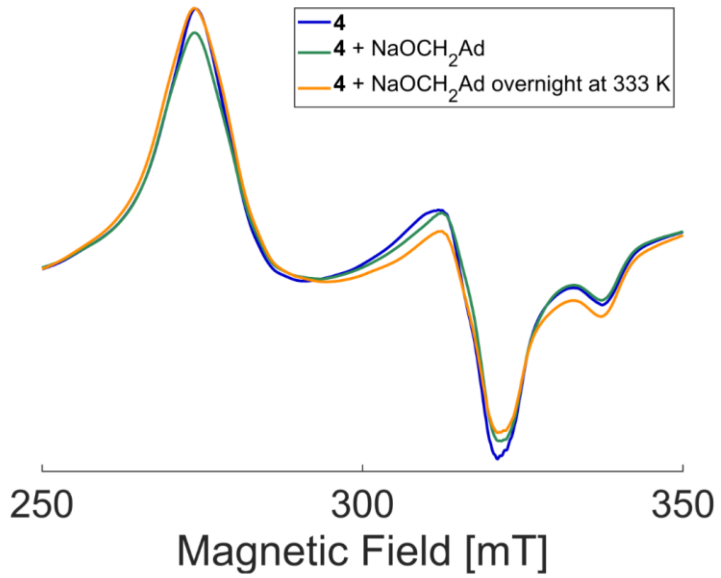

Figure S15: *Experimental X-band EPR spectra of **4** (~30 mM) recorded at 93 K immediately after sample preparation (blue trace), after mixing with $NaOCH_2Ad$ (green trace) and heating the mixture overnight (brown trace) at 333 K. Inspection of the relative EPR intensities shows that 88% of the signal is preserved even after overnight thermal treatment.*

NMR

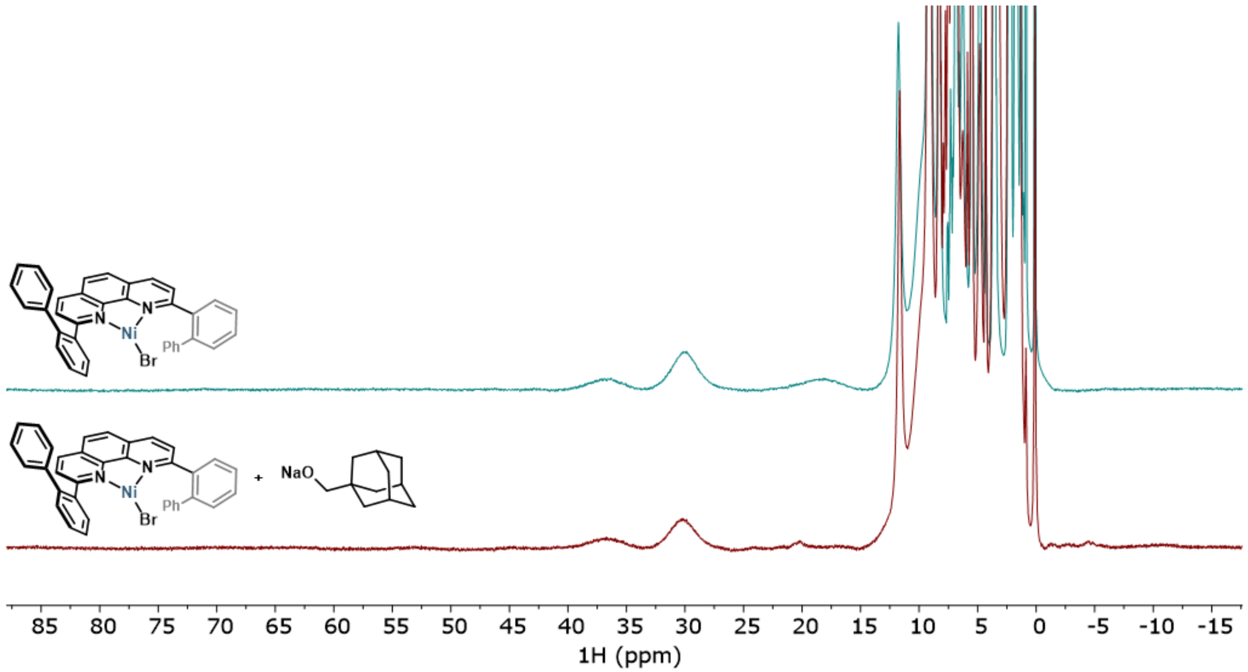

Figure S16: *^{1}H NMR (400 MHz, THF-d_8, 25 °C) of the mixture of* ***4*** *with $NaOCH_2Ad$ (red trace). ^{1}H NMR of* ***4*** *(400 MHz, THF-d_8, 25 °C, teal trace) included for comparison.*

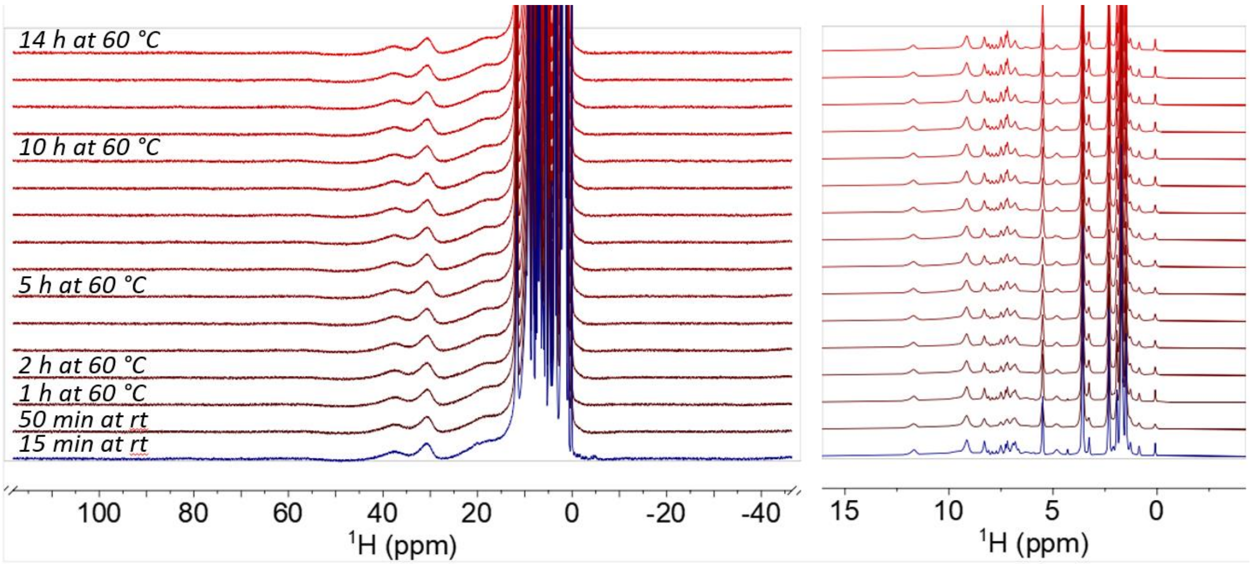

Figure S17*: ^{1}H NMR spectra (400 MHz, THF-d_8, 30 mM, 25 °C) of the mixture of* ***4*** *with $NaOCH_2Ad$ heated at 60 °C over time.*

According to both EPR and NMR analyses, as well as the analysis of the organic products after acidic quench by GC-MS, the salt metathesis does not seem to proceed in this direction, leaving **4** as the dominant nickel species.

8. Oxidation of 4 under N_2O

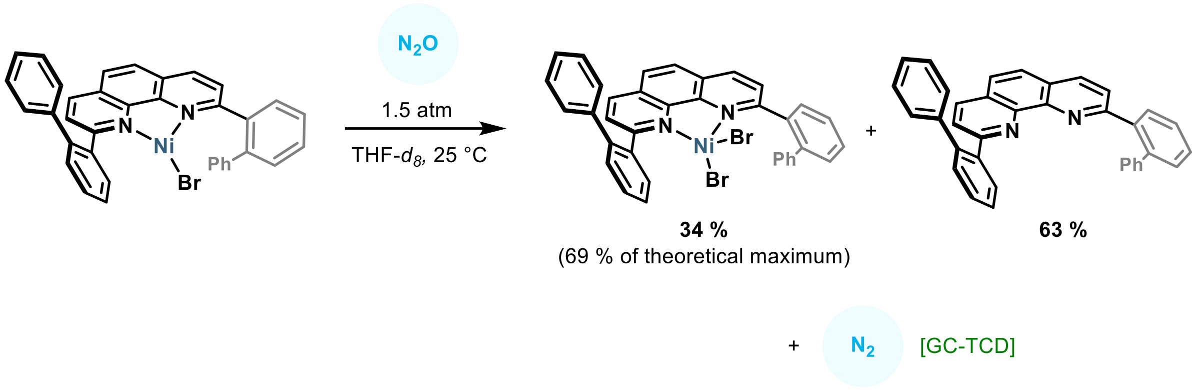

For NMR and EPR analyses, in an argon-filled glovebox, **4** (20 mg, 28 µmol, 1 equiv.) was dissolved in degassed THF-d_8 (1 mL) in an oven-dried glass vial, and 0.5 mL of that solution transferred into a heatgun-dried J-Young NMR tube. Subsequently, the sample in the tube was frozen in liquid nitrogen, and the headspace of the tube evacuated. The J-Young NMR tube was then closed, and the sample was thawed. Using a three-way key, the tube was refilled with N_2O (1.5 atm), and the tube was closed again and inverted once. For EPR analysis, the sample was refrozen in liquid nitrogen after 10 min and measured. For NMR analysis, a ^{1}H NMR spectrum of the sample was recorded after 5 min, and then every 5 min for 1 h 20 min.

EPR

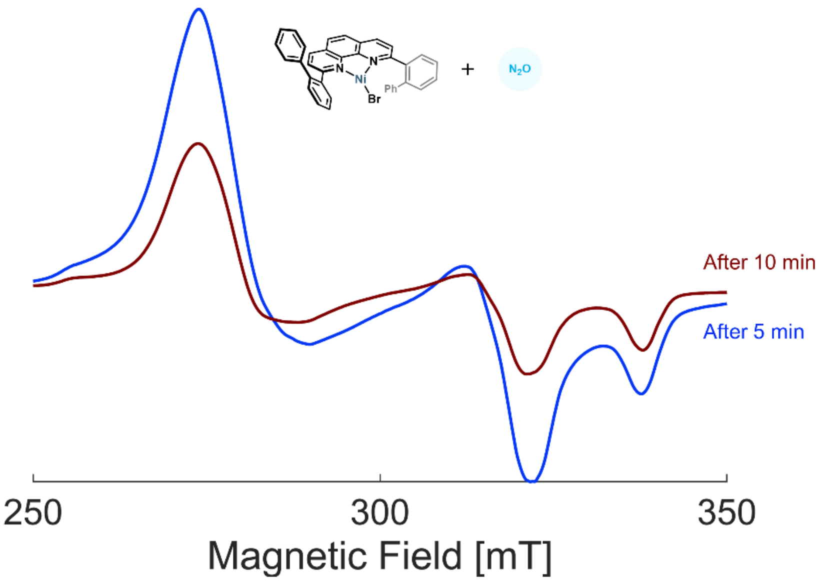

Figure S18: *Comparison of experimental X-band CW-EPR spectrum of **4** in presence of N_2O after 5 minutes and after 10 minutes measured in a frozen solution of THF-d_8 at 93 K.*

NMR

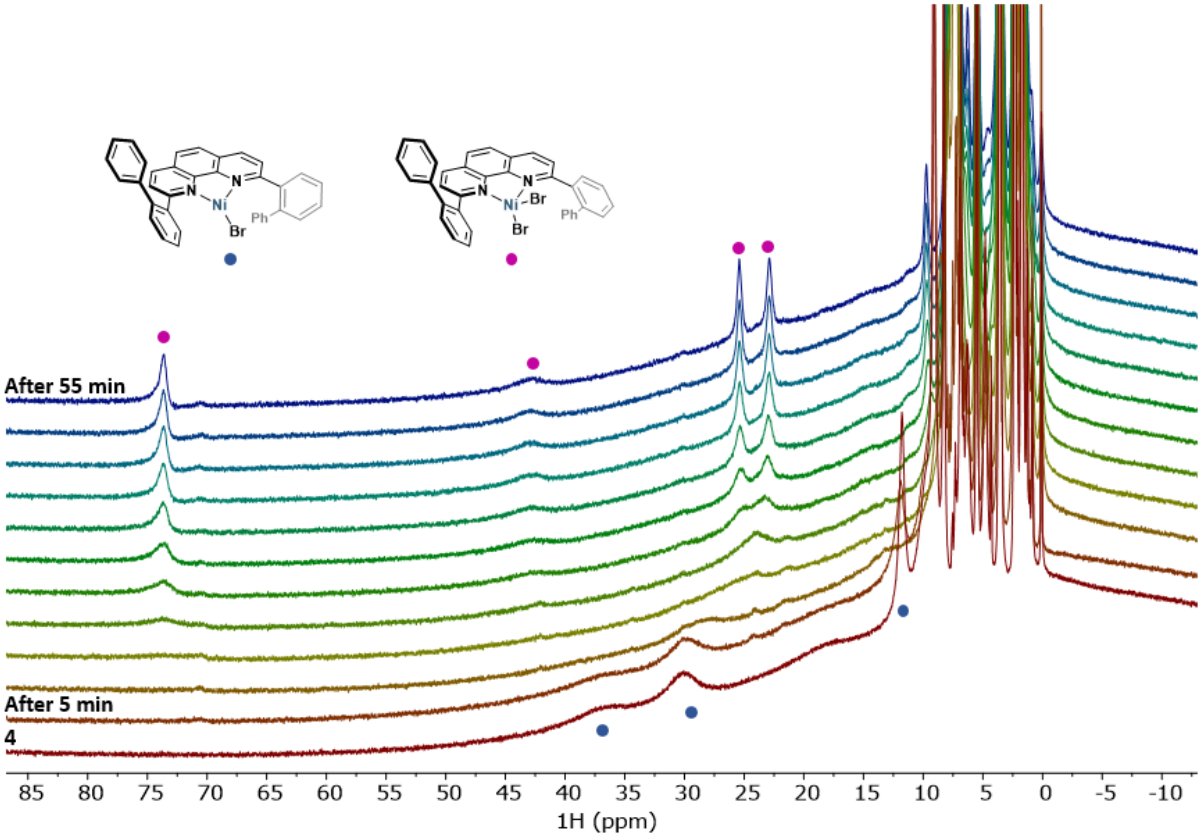

Figure S19: *^{1}H NMR (400 MHz, THF-d$_8$, 25 °C) measured every 5 min during the reaction under 1.5 atm N_2O. ^{1}H NMR of **4** (400 MHz, THF-d$_8$, 25 °C, lowest trace) included for comparison.*

Based on the NMR experiment, **4** is consumed by the N_2O within approx. 15 min, after which **3** emerges. This oxidation is considerable slower than the reaction of the studied Ni(I)alkyl with N_2O, which under the same conditions is consumed within 1 min (*vide supra*). No intermediates were observed.

The oxidation of **4** by N_2O was also conducted in the presence of 1 equiv. of trimethoxybenzene as internal standard to quantify the products. After 2 h 15 min under 1.5 atm N_2O, the yield of **4** was found to be 34%, this is 69% of the theoretical maximum of 50%. Free **L3** also appeared, in 63% yield, ensuing a complete ligand mass balance. We presume that NiO is concomitantly formed in this reaction.

For the GC-TCD analysis of the reaction's headspace, **4** (62 mg, 0.10 mmol) was dissolved in degassed THF-*d$_8$* (2 mL) in a Schlenk flask equipped with a septum, and the resulting mixture frozen in liquid nitrogen. The headspace was evacuated, and after re-closing the Schlenk flask, the mixture was allowed to thaw. Using a three-way key, the Schlenk flask was refilled with N_2O (1.5 atm), and closed again. After stirring at ambient temperature overnight a sample of the headspace's gases was taken with a needle through the septum and analyzed *via* GC-TCD.

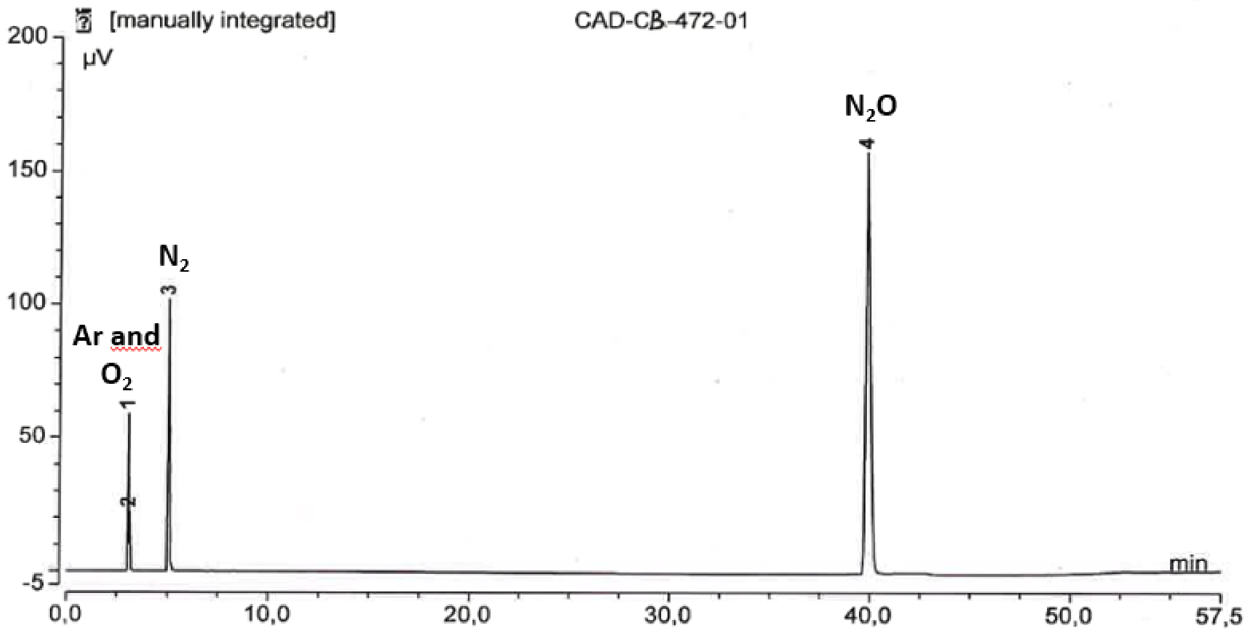

Figure S20: *GC-TCD graph of the headspace of the reaction of **4** with N_2O after overnight stirring at rt, 1.5 atm. Column: 27.7 m, HP-Plot 5 Å Molsieve 0.32/10.0 df G/764. Temperature: 220/30; 10 min iso 6/min 250 12/min 320, 5 min iso/ 250. Gas: 0.50 bar, He. Sample size: 250 μL.*

Table S4: *Peak data of the GC-TCD graph of the headspace of the reaction of **4** with N_2O after overnight stirring at rt, 1.5 atm.*

Peak	Retention time [min]	Area-%	Compound
1	3.06	4.52	Argon (no baseline separation)
2	3.13	1.84	O_2 (no baseline separation)
3	5.04	15.94	N_2
4	39.76	77.71	N_2O

9. Crystallographic Data

Single Crystal structure of 3

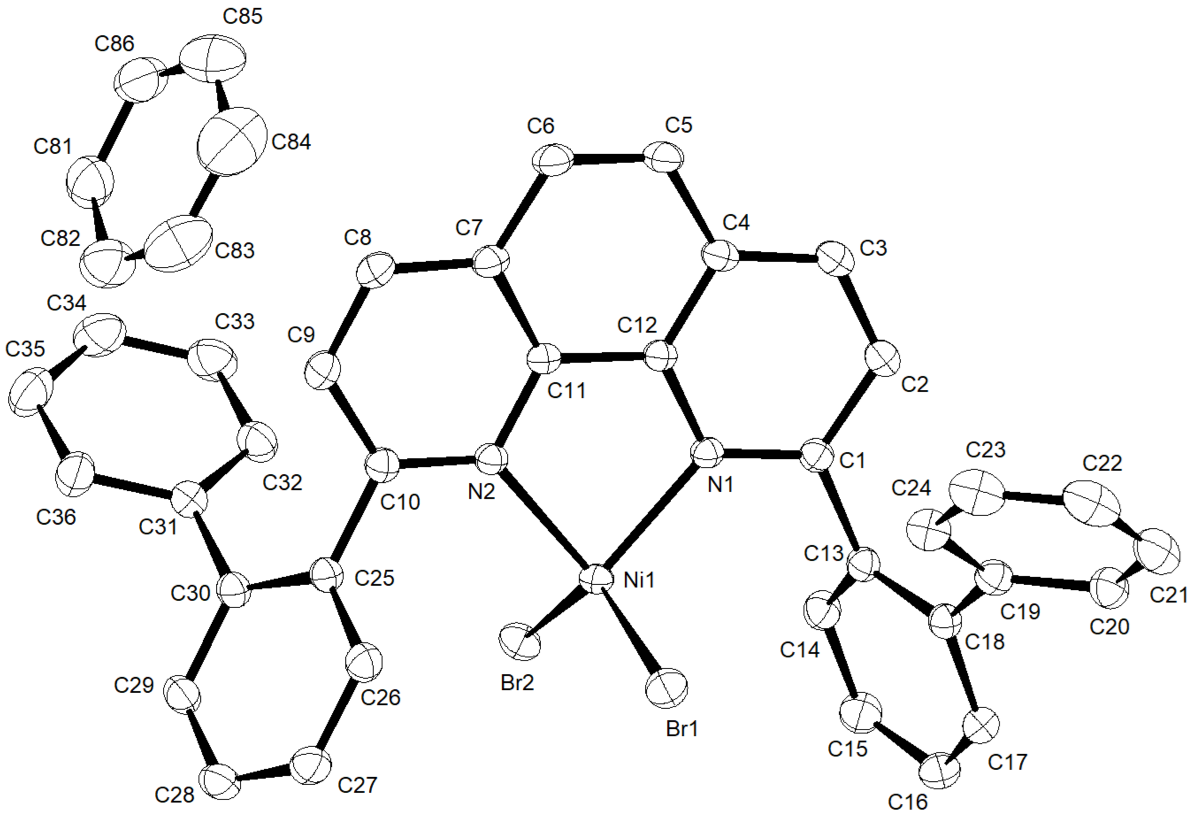

Figure S21. *The molecular structure of* ***3****; H atoms have been removed for clarity.*

X-Ray Crystal Structure Analysis of 3:

$C_{42}H_{30}Br_2N_2Ni$, *Mr* = 781.21 g mol^{-1}, violet block, crystal size 0.195 x 0.179 x 0.105 mm^3, Monoclinic, space group $P2_1/c$ [14], *a* = 12.7498(4) Å, b = 13.9643(4), *c* = 20.3924(5) Å, β = 93.1530(10)°, *V* = 3625.21(18) Å^3, *T* = 150(2) K, *Z* = 4, *Dcalc* = 1.431 g·cm^3, λ = 0.71073 Å, μ(*Mo*-*K*α) = 2.771 mm^{-1}, Gaussian absorption correction (Tmin = 0.70, Tmax = 0.82), Bruker AXS D8-Venture diffractometer with IμS Diamond Mo-anode X-ray source and PHOTON III detector, 2.000 < θ < 33.782°, 851449 measured reflections, 14514 independent reflections, 12601 reflections with $I > 2\sigma(I)$, Rint = 0.0777. The structure was solved by *SHELXT* and refined by full-matrix least-squares (*SHELXL*) against F^2 to $R1$ = 0.023 [$I > 2\sigma(I)$], $wR2$ = 0.063 [all data], 424 parameters and 0 restraints.

Full .cif data for the compound are available under the CCDC number CCDC- 2425233

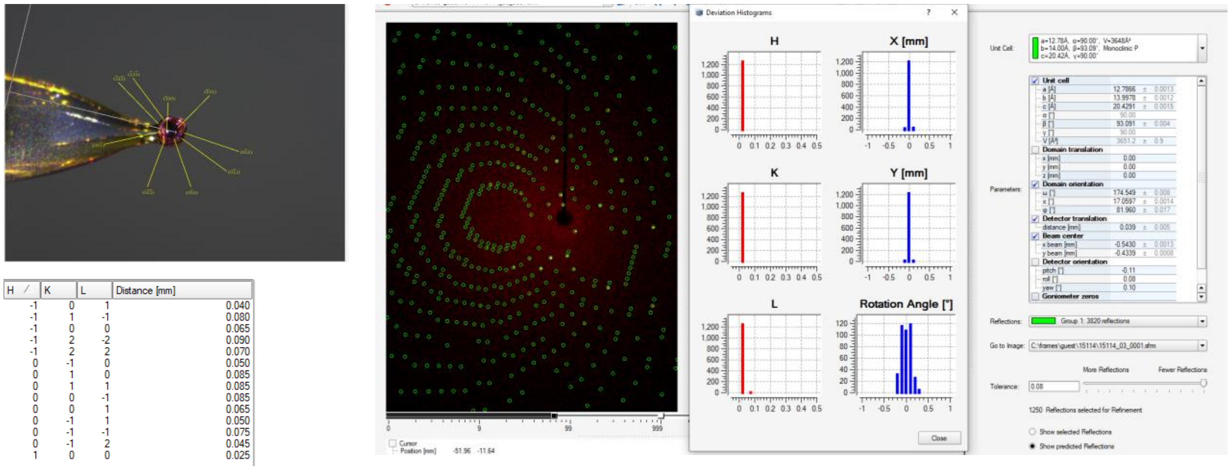

***Figure S22**. Crystal faces and unit cell determination/refinement of **3**.*

***Table S5:** Intensity statistics for dataset of the X-ray crystal structure analysis of **3**.*

Resolution	#Data	#Theory	%Comp	Redundancy	Rrim	Rpim
Inf - 2.66	226	230	98.26	50.04	0.0374	0.0073
2.66 - 1.76	532	532	100.00	79.60	0.0394	0.0053
1.76 - 1.39	756	756	100.00	93.73	0.0419	0.0045
1.39 - 1.21	778	778	100.00	95.68	0.0499	0.0051
1.21 - 1.10	740	740	100.00	86.71	0.0563	0.0060
1.10 - 1.02	748	748	100.00	73.69	0.0654	0.0075
1.02 - 0.96	738	738	100.00	67.26	0.0745	0.0090
0.96 - 0.91	747	747	100.00	62.18	0.0905	0.0114
0.91 - 0.87	780	780	100.00	59.62	0.1080	0.0139
0.87 - 0.83	893	893	100.00	55.42	0.1238	0.0165
0.83 - 0.80	797	797	100.00	54.49	0.1478	0.0199
0.80 - 0.78	607	607	100.00	53.00	0.1467	0.0200
0.78 - 0.75	1022	1022	100.00	50.37	0.1705	0.0239
0.75 - 0.73	766	766	100.00	45.58	0.1952	0.0287

0.73 - 0.72	456	456	100.00	44.55	0.1970	0.0295
0.72 - 0.70	895	895	100.00	43.54	0.2292	0.0347
0.70 - 0.68	1039	1039	100.00	42.35	0.2585	0.0396
0.68 - 0.67	560	560	100.00	39.50	0.2857	0.0453
0.67 - 0.66	610	610	100.00	36.79	0.3452	0.0565
0.66 - 0.65	620	620	100.00	37.61	0.3602	0.0582
0.65 - 0.64	717	740	96.89	33.10	0.3877	0.0654
0.74 - 0.64	5284	5307	99.57	40.17	0.2663	0.0416
Inf - 0.64	15027	15054	99.82	57.67	0.0752	0.0097

Table S6: *Crystal data and structure refinement of* ***3****.*

Identification code	15114	
Empirical formula	$C_{42} H_{30} Br_2 N_2 Ni$	
Color	violet	
Formula weight	781.21 g · mol^{-1}	
Temperature	150(2) K	
Wavelength	0.71073 Å	
Crystal system	MONOCLINIC	
Space group	**$P2_1/c$, (no. 14)**	
Unit cell dimensions	a = 12.7498(4) Å	α = 90°.
	b = 13.9643(4) Å	β = 93.1530(10)°.
	c = 20.3924(5) Å	γ = 90°.
Volume	3625.21(18) Å^3	

Z	4	
Density (calculated)	1.431 $Mg \cdot m^{-3}$	
Absorption coefficient	2.771 mm^{-1}	
F(000)	1576 e	
Crystal size	0.195 x 0.179 x 0.105 mm^3	
θ range for data collection	2.000 to 33.782°.	
Index ranges	$-19 \le h \le 19$, $-21 \le k \le 21$, $-31 \le l \le 30$	
Reflections collected	851449	
Independent reflections	14514 [R_{int} = 0.0777]	
Reflections with $I>2\sigma(I)$	12601	
Completeness to θ = 25.242°	99.9 %	
Absorption correction	Semi-empirical from equivalents	
Max. and min. transmission	0.82 and 0.70	
Refinement method	Full-matrix least-squares on F^2	
Data / restraints / parameters	14514 / 0 / 424	
Goodness-of-fit on F^2	1.020	
Final R indices [I>2□(I)]	R_1 = 0.0230	wR^2 = 0.0589
R indices (all data)	R_1 = 0.0298	wR^2 = 0.0625
Remarks	**'Use solvent mask' was applied!**	
Largest diff. peak and hole	0.6 and -0.5 $e \cdot Å^{-3}$	

Table S7: *Bond lengths [Å] and angles [°] of* ***3****.*

Br(1)-Ni(1)	2.35239(18)	Br(2)-Ni(1)	
2.36434(16)	Ni(1)-N(1)	2.0140(8)	Ni(1)-N(2)
2.0180(8)	N(1)-C(1)	1.3348(12)	N(1)-C(12)
1.3609(12)	N(2)-C(10)	1.3357(12)	N(2)-C(11)
1.3645(12)	C(1)-C(2)	1.4122(13)	C(1)-C(13)
1.4848(13)	C(2)-C(3)	1.3734(15)	C(3)-C(4)
1.4107(15)	C(4)-C(5)	1.4336(14)	C(4)-C(12)
1.4050(13)	C(5)-C(6)	1.3573(15)	C(6)-C(7)
1.4336(14)	C(7)-C(8)	1.4114(15)	C(7)-C(11)
1.4054(13)	C(8)-C(9)	1.3708(15)	C(9)-C(10)
1.4129(14)	C(10)-C(25)	1.4812(14)	C(11)-
C(12)	1.4326(13)	C(13)-C(14)	1.3982(14)
C(13)-C(18)	1.4078(14)	C(14)-C(15)	1.3897(15)
C(15)-C(16)	1.3841(17)	C(16)-C(17)	1.3878(15)
C(17)-C(18)	1.3998(14)	C(18)-C(19)	1.4876(14)
C(19)-C(20)	1.4010(15)	C(19)-C(24)	1.3971(15)
C(20)-C(21)	1.3915(16)	C(21)-C(22)	1.382(2)
C(22)-C(23)	1.386(2)	C(23)-C(24)	1.3901(16)
C(25)-C(26)	1.3960(15)	C(25)-C(30)	1.4064(14)
C(26)-C(27)	1.3919(15)	C(27)-C(28)	1.3843(17)
C(29)-C(30)	1.3987(14)	C(30)-C(31)	1.4860(15)
C(31)-C(32)	1.3954(15)	C(31)-C(36)	1.3962(15)
C(32)-C(33)	1.3913(17)	C(33)-C(34)	1.384(2)
C(34)-C(35)	1.384(2)	C(35)-C(36)	1.3874(18)
C(81)-C(82)	1.375(2)	C(81)-C(86)	1.372(2)
C(82)-C(83)	1.372(3)	C(83)-C(84)	1.383(3)
C(84)-C(85)	1.403(3)	C(85)-C(86)	1.374(3)

Br(1)-Ni(1)-Br(2)	127.642(7)	N(1)-Ni(1)-Br(1)	100.29(2)
N(1)-Ni(1)-Br(2)	119.94(2)	N(1)-Ni(1)-N(2)	82.77(3)
N(2)-Ni(1)-Br(1)	112.71(2)	N(2)-Ni(1)-Br(2)	104.81(2)
C(1)-N(1)-Ni(1)	128.83(6)	C(1)-N(1)-C(12)	119.16(8)

C(12)-N(1)-Ni(1)	111.84(6)	C(10)-N(2)-Ni(1)	129.45(7)
C(10)-N(2)-C(11)	118.97(8)	C(11)-N(2)-Ni(1)	111.58(6)
N(1)-C(1)-C(2)	120.95(9)	N(1)-C(1)-C(13)	116.57(8)
C(2)-C(1)-C(13)	122.46(9)	C(3)-C(2)-C(1)	120.21(9)
C(2)-C(3)-C(4)	119.55(9)	C(3)-C(4)-C(5)	124.04(9)
C(12)-C(4)-C(3)	116.91(9)	C(12)-C(4)-C(5)	119.05(9)
C(6)-C(5)-C(4)	121.09(9)	C(5)-C(6)-C(7)	120.88(9)
C(8)-C(7)-C(6)	123.91(9)	C(11)-C(7)-C(6)	119.19(9)
C(11)-C(7)-C(8)	116.90(9)	C(9)-C(8)-C(7)	119.42(9)
C(8)-C(9)-C(10)	120.55(9)	N(2)-C(10)-C(9)	120.85(9)
N(2)-C(10)-C(25)	118.46(8)	C(9)-C(10)-C(25)	120.64(9)
N(2)-C(11)-C(7)	123.26(9)	N(2)-C(11)-C(12)	116.90(8)
C(7)-C(11)-C(12)	119.83(8)	N(1)-C(12)-C(4)	123.15(9)
N(1)-C(12)-C(11)	116.90(8)	C(4)-C(12)-C(11)	119.95(8)
C(14)-C(13)-C(1)	117.47(9)	C(14)-C(13)-C(18)	120.16(9)
C(18)-C(13)-C(1)	122.37(9)	C(15)-C(14)-C(13)	120.71(10)
C(16)-C(15)-C(14)	119.59(10)	C(15)-C(16)-C(17)	119.98(10)
C(16)-C(17)-C(18)	121.71(10)	C(13)-C(18)-C(19)	123.57(9)
C(17)-C(18)-C(13)	117.82(9)	C(17)-C(18)-C(19)	118.60(9)
C(20)-C(19)-C(18)	119.15(9)	C(24)-C(19)-C(18)	122.26(9)
C(24)-C(19)-C(20)	118.54(10)	C(21)-C(20)-C(19)	120.45(11)
C(22)-C(21)-C(20)	120.42(12)	C(21)-C(22)-C(23)	119.63(11)
C(22)-C(23)-C(24)	120.47(12)	C(23)-C(24)-C(19)	120.48(11)
C(26)-C(25)-C(10)	118.52(9)	C(26)-C(25)-C(30)	120.18(9)
C(30)-C(25)-C(10)	121.21(9)	C(27)-C(26)-C(25)	120.21(10)
C(28)-C(27)-C(26)	120.05(10)	C(27)-C(28)-C(29)	119.88(10)
C(28)-C(29)-C(30)	121.33(10)	C(25)-C(30)-C(31)	122.62(9)
C(29)-C(30)-C(25)	118.33(9)	C(29)-C(30)-C(31)	119.05(9)
C(32)-C(31)-C(30)	121.63(9)	C(32)-C(31)-C(36)	118.95(10)
C(36)-C(31)-C(30)	119.38(9)	C(33)-C(32)-C(31)	120.19(11)
C(34)-C(33)-C(32)	120.23(12)	C(33)-C(34)-C(35)	120.03(12)
C(34)-C(35)-C(36)	120.00(12)	C(35)-C(36)-C(31)	120.59(11)
C(86)-C(81)-C(82)	121.18(16)	C(83)-C(82)-C(81)	119.80(16)
C(82)-C(83)-C(84)	120.02(17)	C(83)-C(84)-C(85)	119.72(18)
C(86)-C(85)-C(84)	119.59(18)	C(81)-C(86)-C(85)	119.68(16)

Single Crystal structure of 4

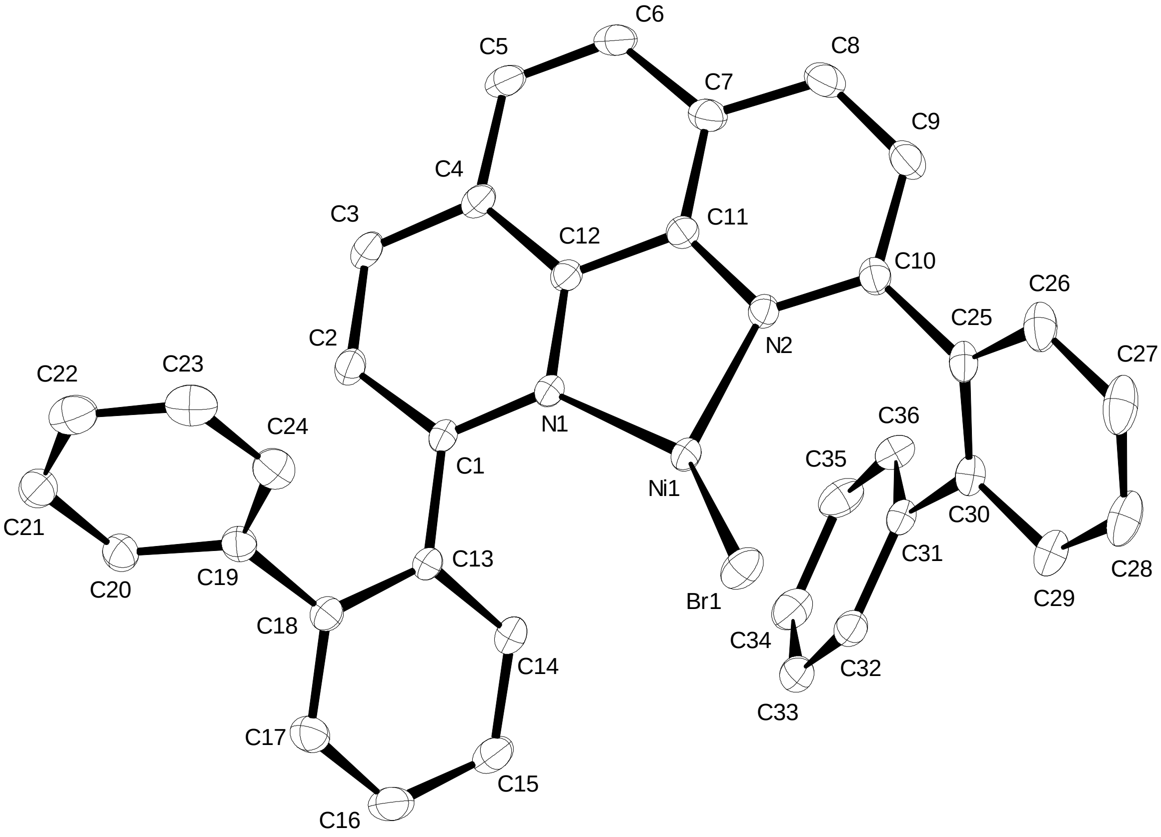

Figure S23: *The molecular structure of **4**; H atoms have been removed for clarity.*

X-ray Crystal Structure Analysis of 4:

$C_{36}H_{24}BrN_2Ni$, *Mr* = 623.19 g mol^{-1}, black prism, crystal size 0.114 x 0.102 x 0.041 mm^3, Monoclinic, space group $P2_1/c$ [14], *a* = 10.0266(3) Å, b = 24.4357(8) Å, *c* = 11.9416(4) Å, □ = 109.8530(10)°, *V* = 2751.89(15) $Å^3$, *T* = 100(2) K, *Z* = 4, *Dcalc* = 1.504 g·cm^3, □ = 0.71073 Å, □(*Mo*-*K*□) = 2.186 mm^{-1}, Gaussian absorption correction (*T*min = 0.83, *T*max = 0.93), Bruker AXS D8-Venture diffractometer with IµS Diamond Mo-anode X-ray source and PHOTON III detector, 1.996 < □ < 30.506°, 377828 measured reflections, 8405 independent reflections, 7784 reflections with *I* > 2σ(*I*), *R*int = 0.0431. The structure was solved by *SHELXT* and refined by full-matrix least-squares (*SHELXL*) against F^2 to *R1* = 0.034 [*I* > 2 σ(*I*)], *wR2* = 0.094 [all data], 361 parameters and 0 restraints.

Full .cif data for the compound are available under the CCDC number CCDC- 2425234

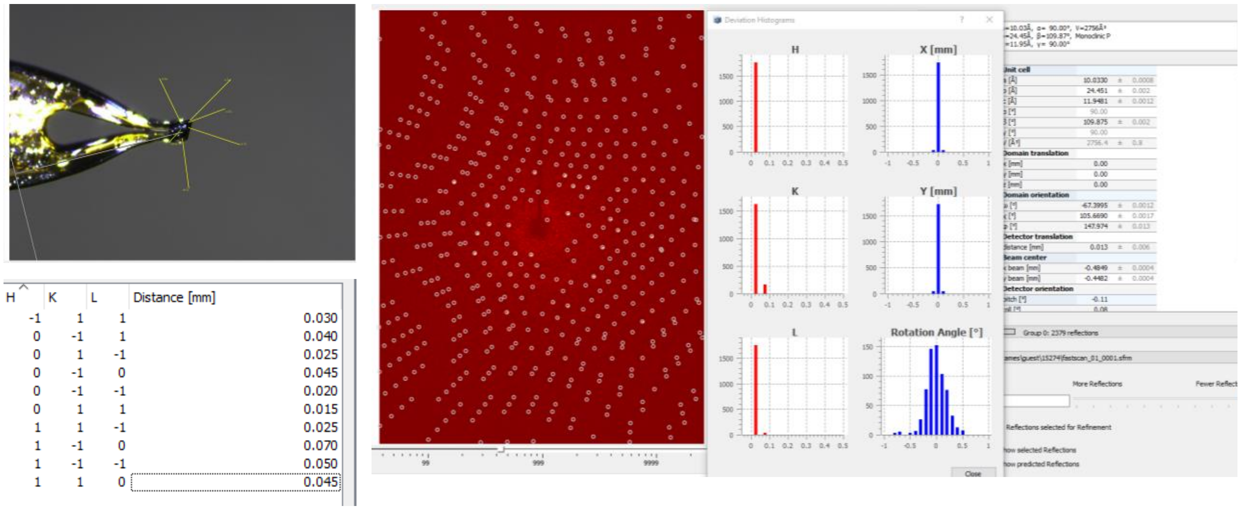

Figure S24: *Crystal faces and unit cell determination/refinement of* ***4.***

Table S8: *Intensity statistics for dataset of the X-ray crystal structure analysis of* ***4***.

Resolution	#Data	#Theory	%Complete	Redundancy	Mean I	Mean I/s	Rmerge	Rsigma
Inf - 2.62	623	642	97.0	13.70	85.83	92.28	0.0238	0.0264
2.62 - 1.76	1471	1474	99.8	16.25	51.38	95.95	0.0210	0.0084
1.76 - 1.40	2090	2090	100.0	16.11	31.18	75.99	0.0246	0.0095
1.40 - 1.22	2150	2150	100.0	16.17	18.96	58.64	0.0328	0.0120
1.22 - 1.11	2078	2078	100.0	15.84	19.68	57.37	0.0355	0.0126
1.11 - 1.03	2140	2144	99.8	15.37	13.72	47.11	0.0448	0.0160
1.03 - 0.97	2063	2068	99.8	12.69	11.76	38.54	0.0498	0.0202
0.97 - 0.92	2099	2104	99.8	11.45	9.84	31.99	0.0548	0.0237
0.92 - 0.88	2179	2192	99.4	10.30	8.26	26.73	0.0640	0.0299
0.88 - 0.85	1770	1780	99.4	10.05	6.16	21.86	0.0778	0.0372
0.85 - 0.82	2177	2204	98.8	9.56	5.65	19.72	0.0865	0.0423
0.82 - 0.79	2429	2456	98.9	9.22	5.31	17.94	0.0915	0.0474
0.79 - 0.77	1865	1884	99.0	8.87	4.59	15.07	0.1021	0.0544
0.77 - 0.75	1956	2006	97.5	8.63	4.28	14.20	0.1123	0.0611
0.75 - 0.73	2371	2406	98.5	7.90	3.98	12.30	0.1279	0.0705
0.73 - 0.71	2451	2508	97.7	7.14	3.73	10.63	0.1401	0.0827

0.71 - 0.70	1401	1442	97.2	6.97	3.56	10.01	0.1479	0.0895
0.70 - 0.68	2978	3050	97.6	7.00	2.84	8.24	0.1743	0.1091
0.68 - 0.67	1544	1576	98.0	6.78	2.77	7.83	0.1873	0.1166
0.67 - 0.66	1736	1822	95.3	5.01	2.48	6.12	0.1947	0.1514
0.66 - 0.65	1902	2110	90.1	3.57	2.60	5.48	0.1844	0.1711
--								
0.75 - 0.65	14383	14914	96.4	6.41	3.17	8.82	0.1562	0.1051
Inf - 0.65	41473	42186	98.3	10.22	11.25	29.33	0.0439	0.0278

Table S9: *Crystal data and structure refinement of* ***Ni(Phen(BiPh)$_2$)Br****.*

Identification code	15274	
Empirical formula	$C_{36} H_{24} Br N_2 Ni$	
Color	black	
Formula weight	623.19 g · mol^{-1}	
Temperature	100(2) K	
Wavelength	0.71073 Å	
Crystal system	MONOCLINIC	
Space group	**$P2_1/c$, (no. 14)**	
Unit cell dimensions	a = 10.0266(3) Å	α = 90°.
	b = 24.4357(8) Å	β = 109.8530(10)°.
	c = 11.9416(4) Å	γ = 90°.
Volume	2751.89(15) Å^3	
Z	4	
Density (calculated)	1.504 Mg · m^{-3}	
Absorption coefficient	2.186 mm^{-1}	
F(000)	1268 e	

Crystal size	0.114 x 0.102 x 0.041 mm^3
θ range for data collection	1.996 to 30.506°.
Index ranges	$-14 \leq h \leq 14$, $-34 \leq k \leq 34$, $-17 \leq l \leq 17$
Reflections collected	377828
Independent reflections	8405 [R_{int} = 0.0431]
Reflections with $I>2\sigma(I)$	7784
Completeness to θ = 25.242°	100.0 %
Absorption correction	Semi-empirical from equivalents
Max. and min. transmission	0.93 and 0.83
Refinement method	Full-matrix least-squares on F^2
Data / restraints / parameters	8405 / 0 / 361
Goodness-of-fit on F^2	1.109
Final R indices [$I>2\sigma(I)$]	R_1 = 0.0343 wR^2 = 0.0923
R indices (all data)	R_1 = 0.0370 wR^2 = 0.0941
Largest diff. peak and hole	1.4 and -0.7 e · $Å^{-3}$

Table S10: *Bond lengths [Å] and angles [°] of **4**.*

Br(1)-Ni(1)	2.2900(3)	Ni(1)-N(1)	1.9587(13)
Ni(1)-N(2)	1.9742(14)	N(1)-C(1)	1.340(2)
N(1)-C(12)	1.3685(19)	N(2)-C(11)	1.365(2)
N(2)-C(10)	1.341(2)	C(1)-C(13)	1.484(2)
C(1)-C(2)	1.415(2)	C(13)-C(14)	1.399(2)
C(13)-C(18)	1.409(2)	C(14)-C(15)	1.390(2)
C(15)-C(16)	1.389(3)	C(16)-C(17)	1.390(3)
C(17)-C(18)	1.401(2)	C(18)-C(19)	1.484(2)
C(19)-C(20)	1.395(2)	C(19)-C(24)	1.401(2)
C(20)-C(21)	1.394(2)	C(21)-C(22)	1.386(3)
C(22)-C(23)	1.390(3)	C(23)-C(24)	1.389(3)
C(2)-C(3)	1.375(2)	C(3)-C(4)	1.410(2)
C(4)-C(12)	1.403(2)	C(4)-C(5)	1.433(2)
C(12)-C(11)	1.431(2)	C(11)-C(7)	1.404(2)
C(10)-C(9)	1.412(2)	C(10)-C(25)	1.483(2)
C(9)-C(8)	1.376(3)	C(8)-C(7)	1.408(2)
C(7)-C(6)	1.434(2)	C(6)-C(5)	1.356(3)
C(25)-C(26)	1.404(2)	C(25)-C(30)	1.407(2)
C(26)-C(27)	1.390(3)	C(27)-C(28)	1.385(3)
C(28)-C(29)	1.390(3)	C(29)-C(30)	1.404(2)
C(30)-C(31)	1.490(2)	C(31)-C(32)	1.399(2)
C(31)-C(36)	1.398(2)	C(32)-C(33)	1.390(2)
C(33)-C(34)	1.394(3)	C(34)-C(35)	1.393(3)
C(35)-C(36)	1.393(2)		

N(1)-Ni(1)-Br(1)	142.86(4)	N(1)-Ni(1)-N(2)	84.24(6)
N(2)-Ni(1)-Br(1)	126.60(4)	C(1)-N(1)-Ni(1)	131.04(11)
C(1)-N(1)-C(12)	118.08(13)	C(12)-N(1)-Ni(1)	110.34(10)
C(11)-N(2)-Ni(1)	110.25(10)	C(10)-N(2)-Ni(1)	130.99(12)
C(10)-N(2)-C(11)	118.05(14)	N(1)-C(1)-C(13)	118.58(13)
N(1)-C(1)-C(2)	121.27(14)	C(2)-C(1)-C(13)	119.81(14)
C(14)-C(13)-C(1)	117.24(15)	C(14)-C(13)-C(18)	119.72(15)

C(18)-C(13)-C(1)	122.57(14)	C(15)-C(14)-C(13)	120.94(17)
C(16)-C(15)-C(14)	119.54(17)	C(15)-C(16)-C(17)	119.98(17)
C(16)-C(17)-C(18)	121.30(17)	C(13)-C(18)-C(19)	122.44(14)
C(17)-C(18)-C(13)	118.41(16)	C(17)-C(18)-C(19)	119.15(15)
C(20)-C(19)-C(18)	120.71(15)	C(20)-C(19)-C(24)	118.71(16)
C(24)-C(19)-C(18)	120.57(15)	C(21)-C(20)-C(19)	120.70(17)
C(22)-C(21)-C(20)	120.14(18)	C(21)-C(22)-C(23)	119.61(17)
C(24)-C(23)-C(22)	120.47(18)	C(23)-C(24)-C(19)	120.36(17)
C(3)-C(2)-C(1)	120.61(15)	C(2)-C(3)-C(4)	119.02(14)
C(3)-C(4)-C(5)	123.58(15)	C(12)-C(4)-C(3)	117.18(14)
C(12)-C(4)-C(5)	119.22(15)	N(1)-C(12)-C(4)	123.80(14)
N(1)-C(12)-C(11)	116.45(13)	C(4)-C(12)-C(11)	119.73(14)
N(2)-C(11)-C(12)	115.94(14)	N(2)-C(11)-C(7)	124.03(15)
C(7)-C(11)-C(12)	120.02(14)	N(2)-C(10)-C(9)	121.27(15)
N(2)-C(10)-C(25)	118.28(15)	C(9)-C(10)-C(25)	120.43(15)
C(8)-C(9)-C(10)	120.47(16)	C(9)-C(8)-C(7)	119.30(16)
C(11)-C(7)-C(8)	116.85(16)	C(11)-C(7)-C(6)	119.13(15)
C(8)-C(7)-C(6)	124.01(15)	C(5)-C(6)-C(7)	120.84(15)
C(6)-C(5)-C(4)	121.01(15)	C(26)-C(25)-C(10)	117.45(15)
C(26)-C(25)-C(30)	119.85(16)	C(30)-C(25)-C(10)	122.67(14)
C(27)-C(26)-C(25)	120.53(18)	C(28)-C(27)-C(26)	120.00(17)
C(27)-C(28)-C(29)	119.87(18)	C(28)-C(29)-C(30)	121.34(17)
C(25)-C(30)-C(31)	124.01(14)	C(29)-C(30)-C(25)	118.34(15)
C(29)-C(30)-C(31)	117.56(15)	C(32)-C(31)-C(30)	120.10(14)
C(36)-C(31)-C(30)	121.29(15)	C(36)-C(31)-C(32)	118.32(15)
C(33)-C(32)-C(31)	121.00(15)	C(32)-C(33)-C(34)	120.29(16)
C(35)-C(34)-C(33)	119.16(16)	C(36)-C(35)-C(34)	120.50(16)
C(35)-C(36)-C(31)	120.72(16)		

10. DFT Study on the reaction of 5 with N_2O

A DFT study was performed for the crucial mechanistic steps of N_2O activation. The coordination sphere of Ni in reactant **5** can adopt both a “T-shaped” and a “Y-shaped” geometry. Both are minima and the latter is marginally more stable (by 1.3 kcal/mol) and is consistent with the EPR observations, specifically with the *g*-tensor components as discussed above. In terms of the interaction with N_2O, however, the complex needs to adopt the T-shaped geometry in order to present an open coordination site. Therefore, all calculations in the following have used the T-shaped form of **5** as starting structure and as energy reference in order to enable coordination of N_2O. The first question concerns the nature of the initial interaction of N_2O with the Ni center of **5**. This was investigated based on three possible coordination modes: (i) O-bound, where the oxygen atom binds directly to Ni; (ii) N-bound mode, where the terminal nitrogen coordinates with the metal; and (iii) the side-on NN-bound mode, involving the formation of a three-center bond between the metal and both nitrogen atoms of the N_2O molecule (Figure S25).

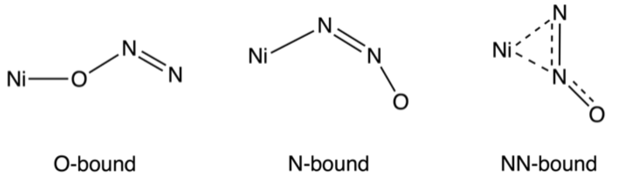

Figure S25: *Three binding modes of N_2O to the Ni center.*

All 3 binding modes for adduct ***add-I*** were identified as distinct minima (Figure S26). All binding modes are endergonic in the gas phase based on the relative Gibbs free energies of the separated reactants, however when the solvent (THF) is taken into account, all binding modes are exergonic. All different binding modes are energetically close to each other with the free binding energy ranging from −6.1 to −2.2 kcal/mol. Crucially, the different coordination modes are clearly differentiated in terms of their electronic structure. This becomes apparent from the comparison of Mulliken spin populations (Table S11), which indicate that incipient oxidation of the Ni^{I} center and concomitant activation of N_2O occurs only in the case of O-bound N_2O. In contrast, the other binding modes do not result in N_2O activation. This result establishes the existence of a specific coordination requirement for subsequent reactivity to unfold. Adduct ***add-I*** (ΔG_{solv} = −3.5 kcal/mol) is chosen for further mechanistic investigation.

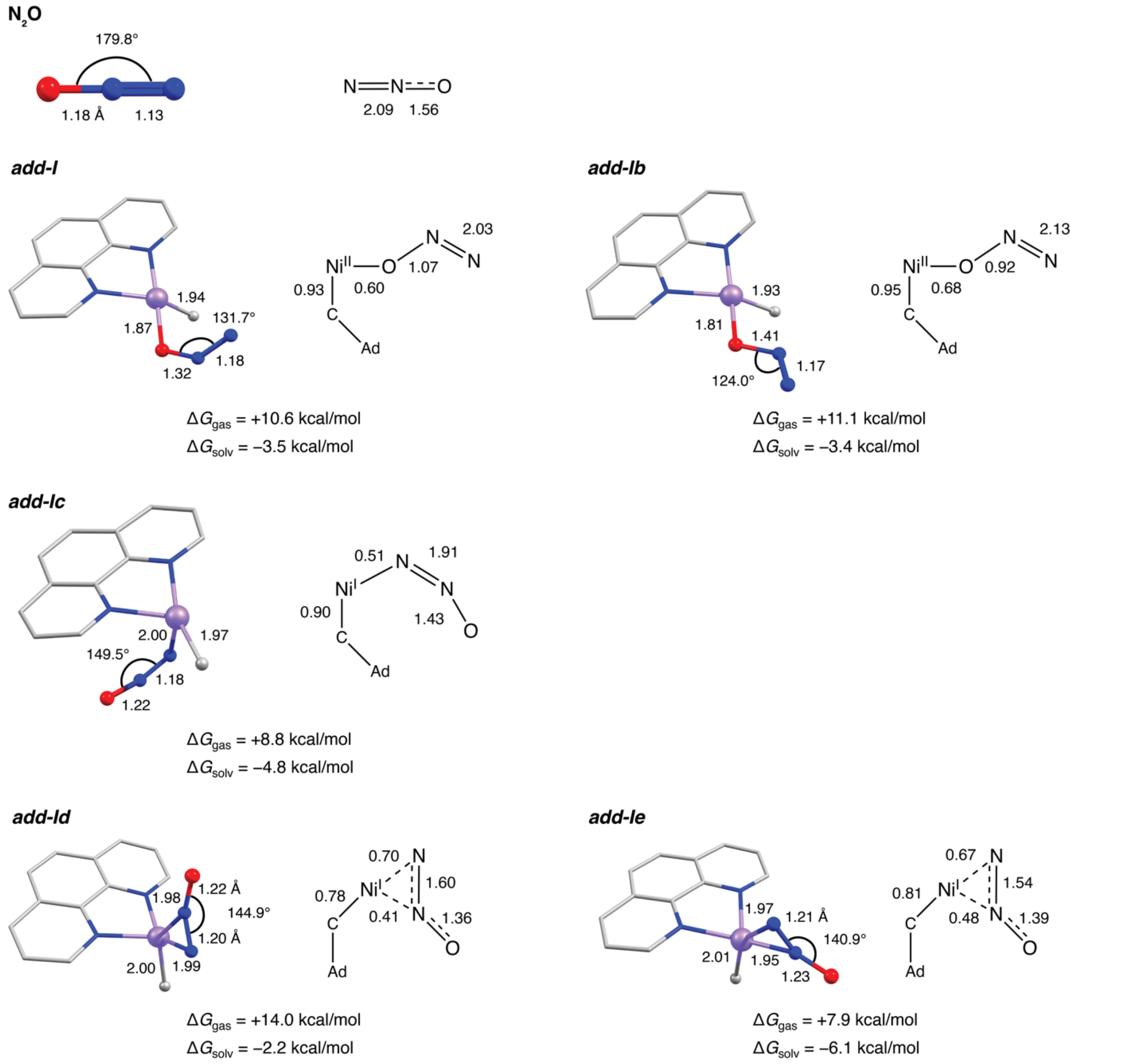

Figure S26: *Different cases of binding modes of N_2O to the Ni center. Geometric parameters (3D representation, large biphenyl and adamantyl groups are hidden for clarity) and Mayer bond orders (2D representation) are shown.*

Table S11: *Mulliken spin populations for the Ni atom and N_2O for the N_2O bound structures.*

Intermediates	Ni	O	N	N
add-I	**0.238**	0.044	0.161	0.615
add-Ib	**0.024**	0.237	0.133	0.599
add-Ic	1.023	-0.036	-0.047	-0.086
add-Id	1.020	-0.051	-0.065	-0.043
add-Ie	1.071	-0.046	-0.101	-0.017

From the O-coordinated adduct, loss of N_2 leads to a new intermediate (***int-Ia***) that is thermodynamically more stable than the reactants ($\Delta G_{solv(2\text{-}3)}$ = −27.0 kcal/mol). There are two possible spin states for ***int-Ia***; a spin doublet (S = 1/2) and a spin quartet (S = 3/2). The former could be formally conceived as a low-spin Ni(III)-oxo form or low-spin Ni(II)-oxyl, whereas the latter as a high-spin Ni(III)-oxo or high spin Ni(II)-oxyl. When calculating the doublet state, significant radical character on the O atom is noticed based on the spin Mulliken populations (see Table S12), implying that the complex is best described as a low-spin Ni^{II}-oxyl, albeit with significant spin delocalization on Ni. Optimization of the spin quartet state results in a high-spin Ni^{II}-oxyl formulation, 6.9 kcal/mol higher in energy than the low-spin case, with the Ni adopting a tetrahedral coordination geometry. Under no condition could a pure oxo species be obtained. Thus, this intermediate is expected to be a spin doublet Ni^{II}-oxyl.

Table S12*: Mulliken spin populations (ρ) for the Ni and O atoms of the two spin states of* ***int-Ia*** *(low-spin, LS, and high-spin HS) and their relative free energies (ΔG) with respect to reactant* ***5*** *in kcal/mol.*

Intermediates	ρ(Ni)	ρ(O)	ΔG
Int-Ia (LS)	0.692	0.505	**-27.0**
Int-Ia (HS)	1.366	0.988	-20.1

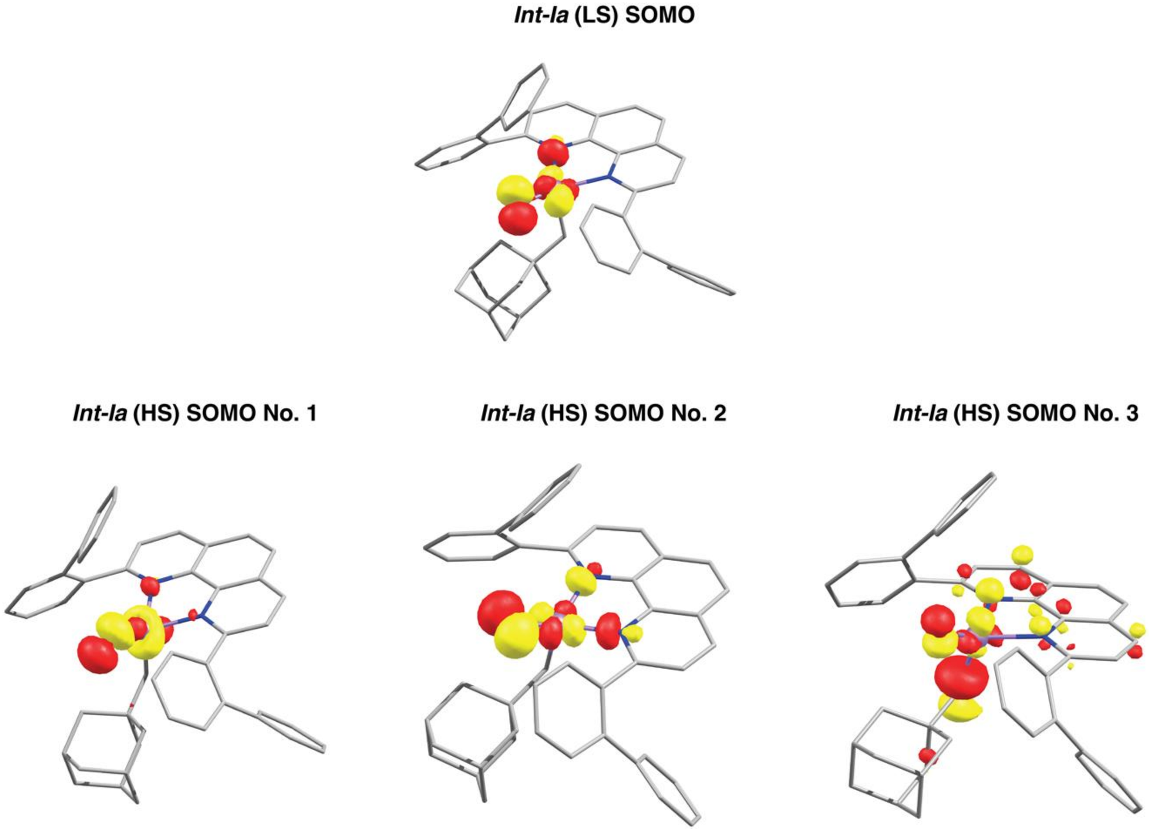

Figure S27*: Graphical representation of the computed Singly Occupied Molecular Orbitals (SOMOs) of the intermediate* ***Int-Ia*** *for the low-spin (LS) state and the high-spin (HS) state.*

This low-spin oxyl form of intermediate ***int-Ia*** is primed to perform O insertion in the Ni-C(Ad) bond. A transition state (**TS-I**) for this was located with a low barrier of 7 kcal/mol and the appropriate imaginary mode corresponding to the concerted breaking of the Ni-C bond and insertion of O (3-membered ring TS). The product ***int-I*** is stabilized by 41.6 kcal/mol (ΔG_{solv} = −68.6 kcal/mol compared to the initial reactants). It is conceivable that a direct pathway may exist from ***add-I*** to ***int-I*** with concerted N_2 loss and O insertion, but despite extensive search, such pathway could not be found for the present system.

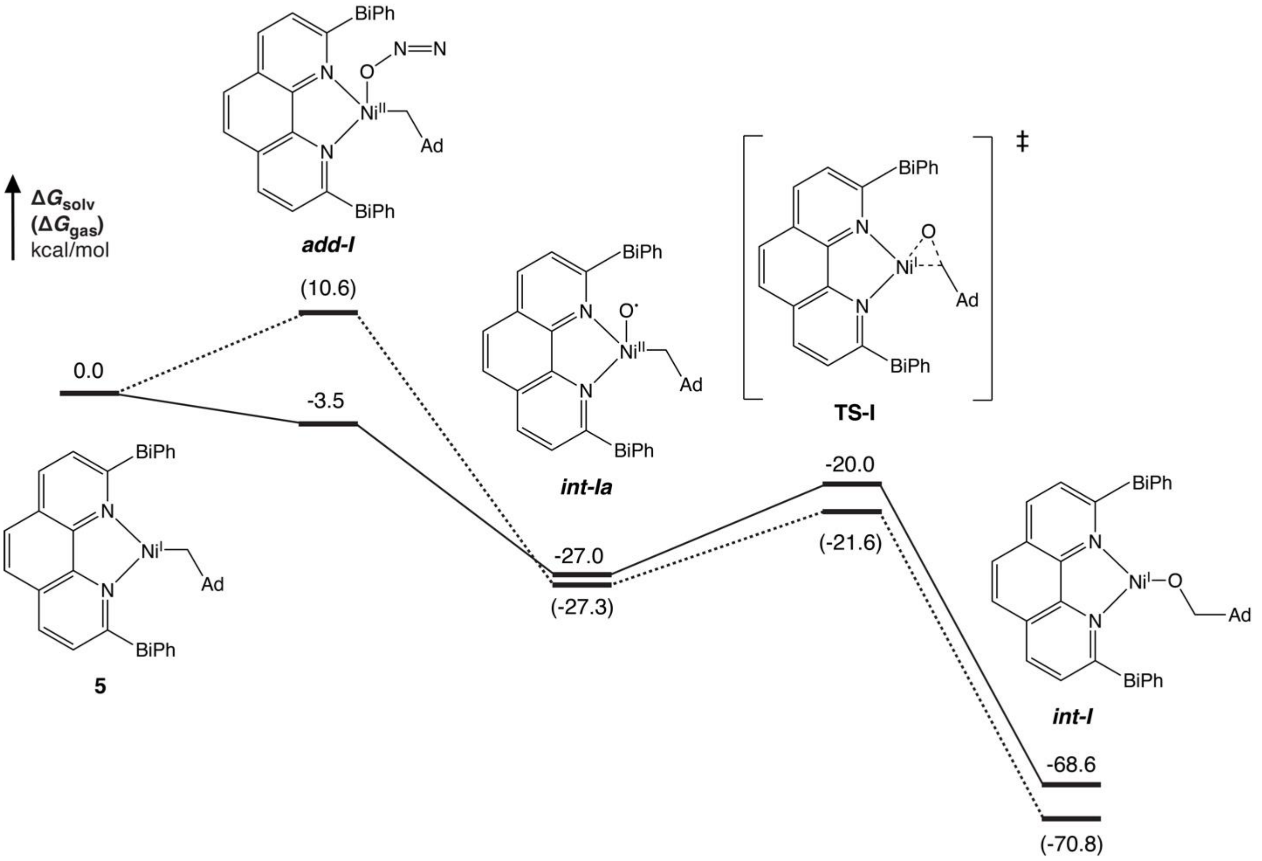

Figure S28*: Proposed reaction pathway from the computational study based on Gibbs free energies (in kcal/mol) in solution and in the gas phase (shown in parenthesis).*

The subsequent metathesis with $MgBr_2$ was calculated to be thermodynamically favorable, with ΔG_{solv} = –9.5 kcal/mol.

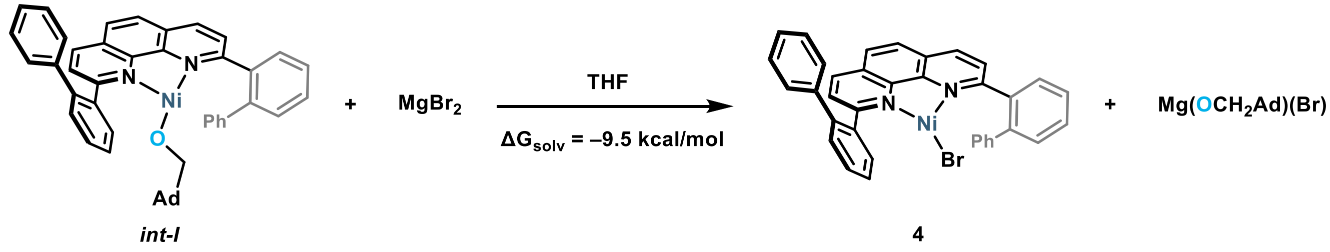

11. References

(1) Jancsó, G. Effect of D and 18O isotope substitution on the absorption spectra of aqueous copper sulfate solutions. *Radiat. Phys. Chem.* **2005**, *74* (3), 168-171. DOI: https://doi.org/10.1016/j.radphyschem.2005.04.011.

(2) Stoll, S.; Schweiger, A. EasySpin, a comprehensive software package for spectral simulation and analysis in EPR. *J. Magn. Reson.* **2006**, *178* (1), 42-55. DOI: https://doi.org/10.1016/j.jmr.2005.08.013.

(3) Neese, F. Software update: The ORCA program system—Version 5.0. *Wiley Interdiscip. Rev.:Comput. Mol. Sci.* **2022**, *12* (5), e1606. DOI: https://doi.org/10.1002/wcms.1606 (acccessed 2025/01/15).

(4) Furness, J. W.; Kaplan, A. D.; Ning, J. L.; Perdew, J. P.; Sun, J. W. Accurate and Numerically Efficient r2SCAN Meta-Generalized Gradient Approximation. *J. Phys. Chem. Lett.* **2020**, *11* (19), 8208-8215, Article. DOI: 10.1021/acs.jpclett.0c02405.

(5) Weigend, F.; Ahlrichs, R. Balanced basis sets of split valence, triple zeta valence and quadruple zeta valence quality for H to Rn: Design and assessment of accuracy. *Phys. Chem. Chem. Phys.* **2005**, *7* (18), 3297-3305, Article. DOI: 10.1039/b508541a.

(6) Caldeweyher, E.; Ehlert, S.; Hansen, A.; Neugebauer, H.; Spicher, S.; Bannwarth, C.; Grimme, S. A generally applicable atomic-charge dependent London dispersion correction. *J. Chem. Phys.* **2019**, *150* (15). DOI: 10.1063/1.5090222 (acccessed 1/27/2025).

(7) Marenich, A. V.; Cramer, C. J.; Truhlar, D. G. Universal Solvation Model Based on Solute Electron Density and on a Continuum Model of the Solvent Defined by the Bulk Dielectric Constant and Atomic Surface Tensions. *J. Phys. Chem. B* **2009**, *113* (18), 6378-6396. DOI: 10.1021/jp810292n.

(8) Ásgeirsson, V.; Birgisson, B. O.; Bjornsson, R.; Becker, U.; Neese, F.; Riplinger, C.; Jónsson, H. Nudged Elastic Band Method for Molecular Reactions Using Energy-Weighted Springs Combined with Eigenvector Following. *J. Chem. Theory Comput.* **2021**, *17* (8), 4929-4945. DOI: 10.1021/acs.jctc.1c00462.

(9) Grimme, S. Semiempirical hybrid density functional with perturbative second-order correlation. *J. Chem. Phys.* **2006**, *124* (3). DOI: 10.1063/1.2148954 (acccessed 1/15/2025).

(10) Neese, F. An improvement of the resolution of the identity approximation for the formation of the Coulomb matrix. *J. Comput. Chem.* **2003**, *24* (14), 1740-1747. DOI: https://doi.org/10.1002/jcc.10318.

(11) Stoychev, G. L.; Auer, A. A.; Neese, F. Automatic Generation of Auxiliary Basis Sets. *J. Chem. Theory Comput.* **2017**, *13* (2), 554-562. DOI: 10.1021/acs.jctc.6b01041.

(12) Sinnecker, S.; Slep, L. D.; Bill, E.; Neese, F. Performance of Nonrelativistic and Quasi-Relativistic Hybrid DFT for the Prediction of Electric and Magnetic Hyperfine Parameters in 57Fe Mössbauer Spectra. *Inorg. Chem.* **2005**, *44* (7), 2245-2254. DOI: 10.1021/ic048609e.

(13) Rega, N.; Cossi, M.; Barone, V. Development and validation of reliable quantum mechanical approaches for the study of free radicals in solution. *J. Chem. Phys.* **1996**, *105* (24), 11060-11067. DOI: 10.1063/1.472906 (acccessed 1/15/2025).

(14) Heß, B. A.; Marian, C. M.; Wahlgren, U.; Gropen, O. A mean-field spin-orbit method applicable to correlated wavefunctions. *Chem. Phys. Lett.* **1996**, *251* (5), 365-371. DOI: https://doi.org/10.1016/0009-2614(96)00119-4.

(15) Jakobsen, S.; Tilset, M. A rapid synthesis of asymmetric alkyl- and aryl-2,9-disubstitued 1,10-phenanthrolines. *Tetrahedron Lett.* **2011**, *52* (24), 3072-3074. DOI: https://doi.org/10.1016/j.tetlet.2011.04.006.
(16) Hu, M.-Y.; He, Q.; Fan, S.-J.; Wang, Z.-C.; Liu, L.-Y.; Mu, Y.-J.; Peng, Q.; Zhu, S.-F. Ligands with 1,10-phenanthroline scaffold for highly regioselective iron-catalyzed alkene hydrosilylation. *Nat. Commun.* **2018**, *9* (1), 221. DOI: 10.1038/s41467-017-02472-6.
(17) Goodman, M. S.; Hamilton, A. D.; Weiss, J. Self-Assembling, Chromogenic Receptors for the Recognition of Dicarboxylic Acids. *J. Am. Chem. Soc.* **1995**, *117* (32), 8447-8455. DOI: 10.1021/ja00137a021.
(18) Kohler, L.; Hayes, D.; Hong, J.; Carter, T. J.; Shelby, M. L.; Fransted, K. A.; Chen, L. X.; Mulfort, K. L. Synthesis, structure, ultrafast kinetics, and light-induced dynamics of CuHETPHEN chromophores. *Dalton Trans.* **2016**, *45* (24), 9871-9883, 10.1039/C6DT00324A. DOI: 10.1039/C6DT00324A.
(19) Wang, T.; Chen, F.; Qin, J.; He, Y.-M.; Fan, Q.-H. Asymmetric Ruthenium-Catalyzed Hydrogenation of 2- and 2,9-Substituted 1,10-Phenanthrolines. *Angew. Chem. Int. Ed.* **2013**, *52* (28), 7172-7176. DOI: https://doi.org/10.1002/anie.201301830.
(20) Yuan, Y.; Sun, N.-X.; Wang, C.-S.; Guo, K.; Wu, X.-F. Cobalt-Catalyzed Hydroxymethylation of Alkyl Halides with CO as the C1 Source. *Org. Lett.* **2023**, *25* (27), 5084-5088. DOI: 10.1021/acs.orglett.3c01811.
(21) Newman-Stonebraker, S. H.; Raab, T. J.; Roshandel, H.; Doyle, A. G. Synthesis of Nickel(I)–Bromide Complexes via Oxidation and Ligand Displacement: Evaluation of Ligand Effects on Speciation and Reactivity. *J. Am. Chem. Soc.* **2023**. DOI: 10.1021/jacs.3c06233.
(22) Mabbs, F. E.; Collison, D. *Electron Paramagnetic Resonance of d Transition Metal Compounds*; Elsevier, 1992.
(23) Dawson, G. A.; Lin, Q.; Neary, M. C.; Diao, T. Ligand Redox Activity of Organonickel Radical Complexes Governed by the Geometry. *J. Am. Chem. Soc.* **2023**, 20551–20561. DOI: 10.1021/jacs.3c07031.
(24) Somerville, R. J.; Odena, C.; Obst, M. F.; Hazari, N.; Hopmann, K. H.; Martin, R. Ni(I)–Alkyl Complexes Bearing Phenanthroline Ligands: Experimental Evidence for CO2 Insertion at Ni(I) Centers. *J. Am. Chem. Soc.* **2020**, *142* (25), 10936-10941. DOI: 10.1021/jacs.0c04695.
(25) Alvarez, S. Bonding and stereochemistry of three-coordinated transition metal compounds. *Coord. Chem. Rev.* **1999**, *193-195*, 13-41. DOI: https://doi.org/10.1016/S0010-8545(99)00085-5.
(26) Lin, H.-S.; Paquette, L. A. A Convenient Method for Determining the Concentration of Grignard Reagents. *Synth. Commun.* **1994**, *24* (17), 2503-2506. DOI: 10.1080/00397919408010560.
(27) Krasovskiy, A.; Knochel, P. Convenient Titration Method for Organometallic Zinc, Magnesium, and Lanthanide- Reagents. *Synthesis* **2006**, *2006* (05), 0890-0891. DOI: 10.1055/s-2006-926345.

11. NMR Spectra of new compounds

Ligands

^{1}H NMR of L4 (400 MHz, $CDCl_3$, 25 °C)

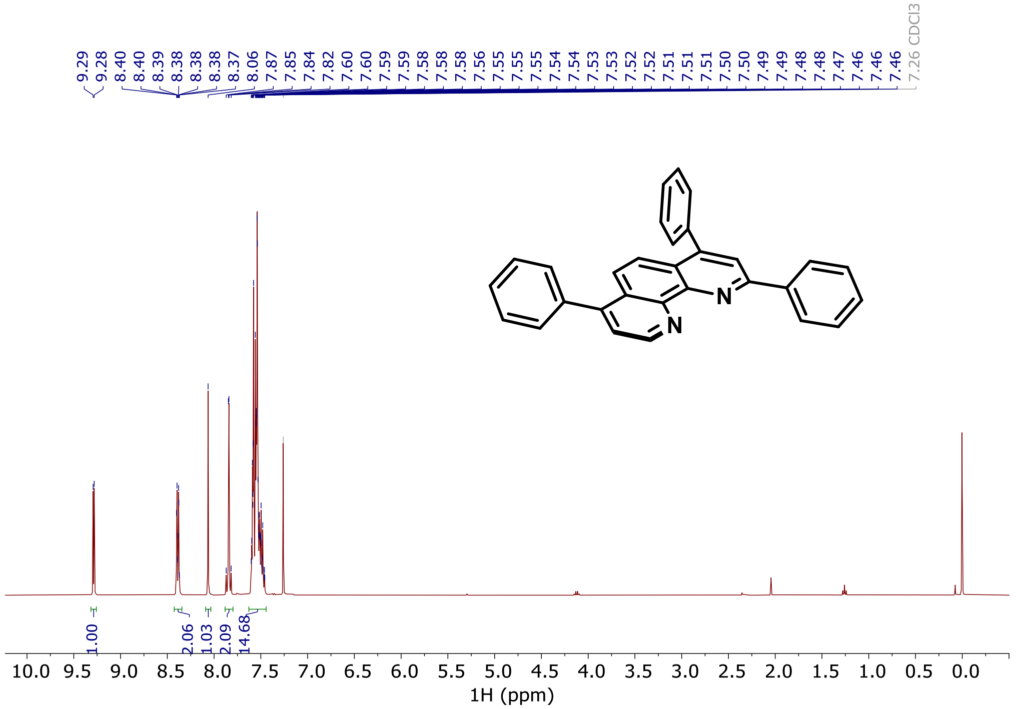

^{13}C NMR of L4 (101 MHz, $CDCl_3$, 25 °C)

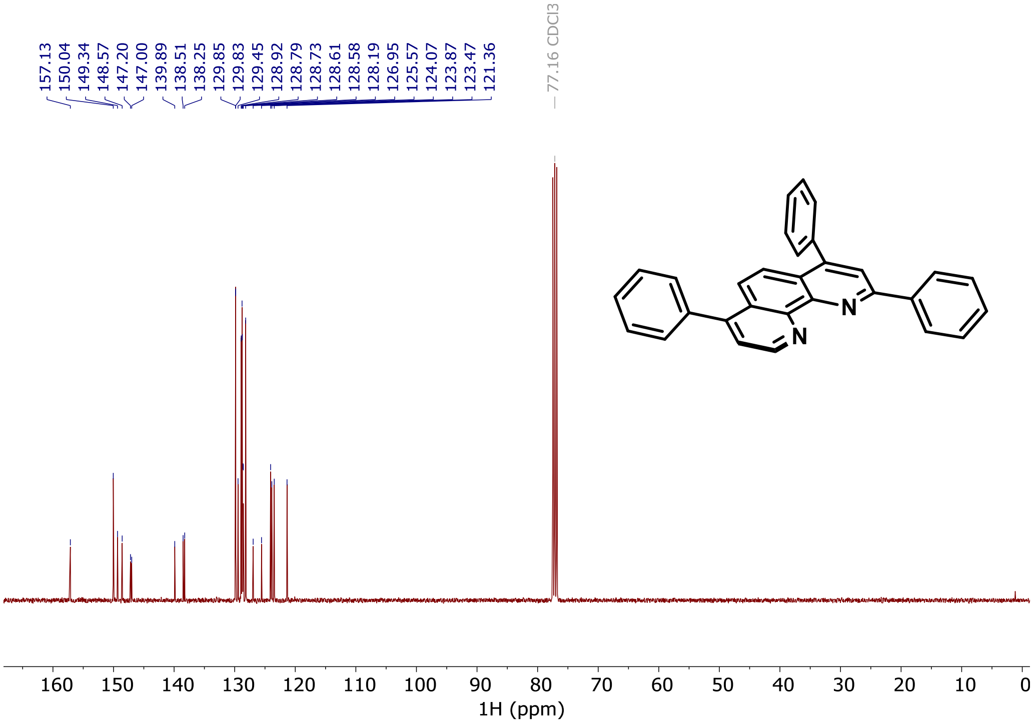

^{1}H NMR of L5 (400 MHz, $CDCl_3$, 25 °C)

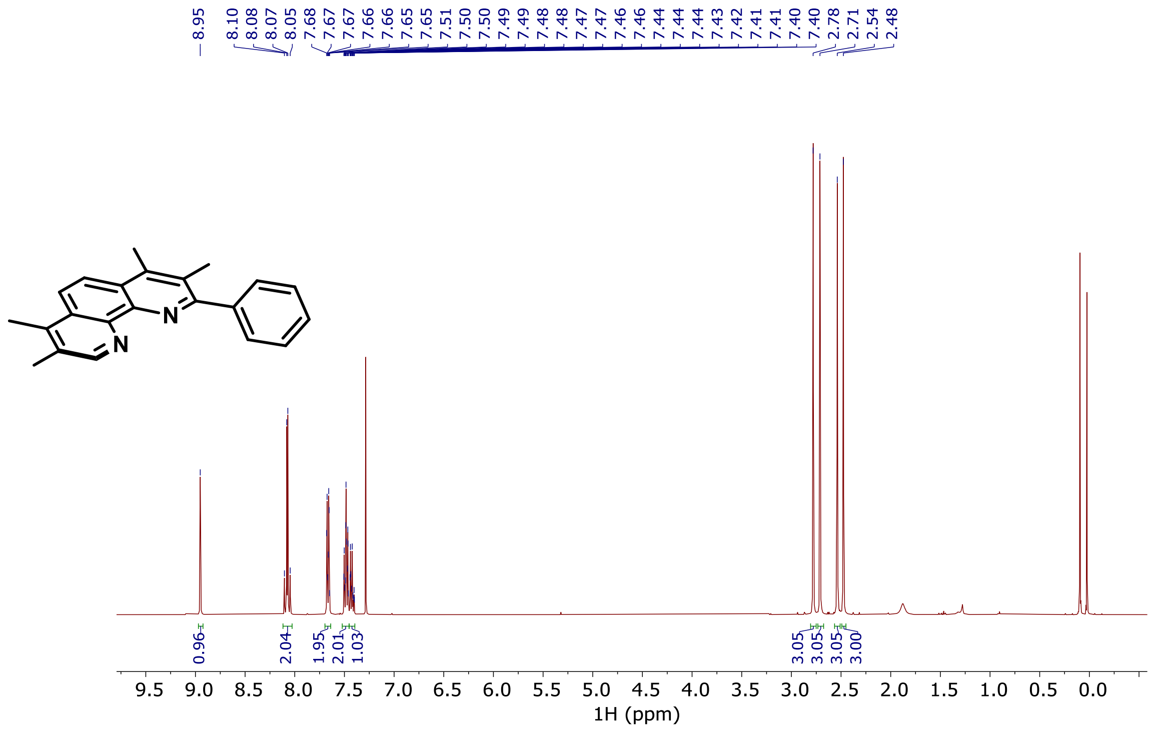

^{13}C NMR of L5 (101 MHz, $CDCl_3$, 25 °C)

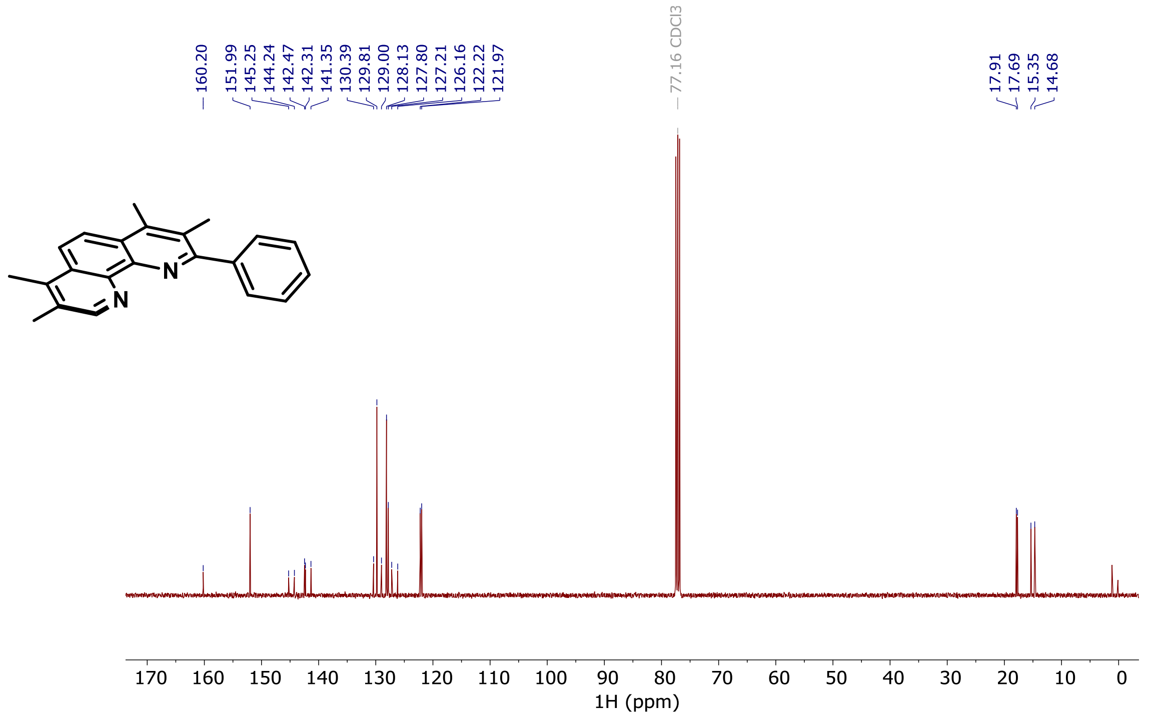

^{1}H NMR of L3 (400 MHz, $CDCl_3$, 25 °C)

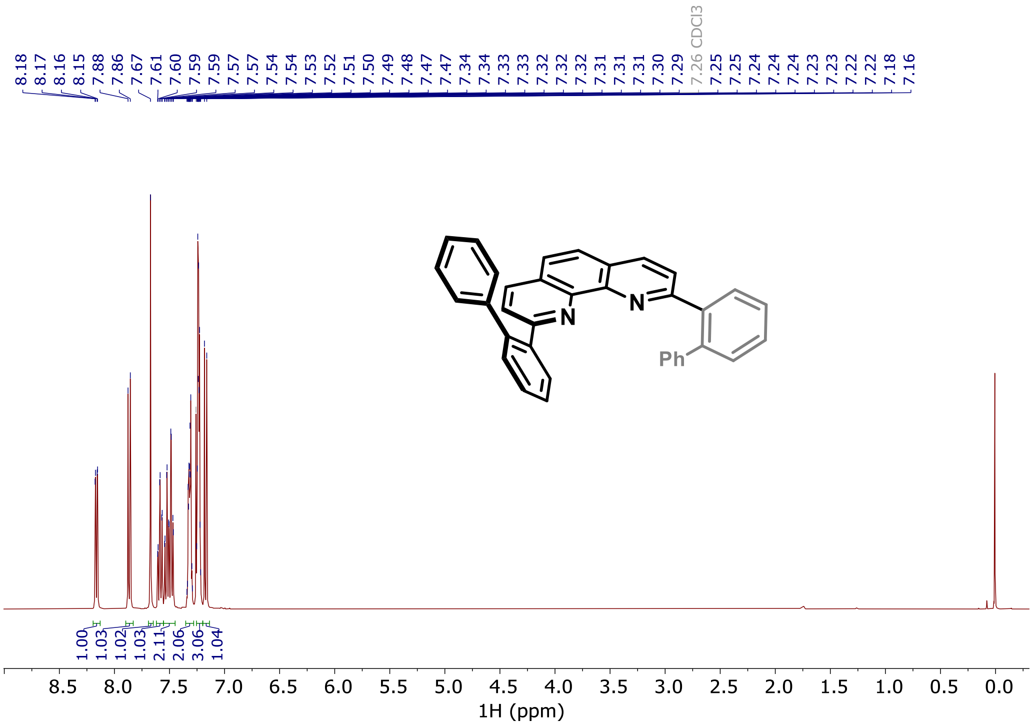

^{13}C NMR of L3 (101 MHz, $CDCl_3$, 25 °C)

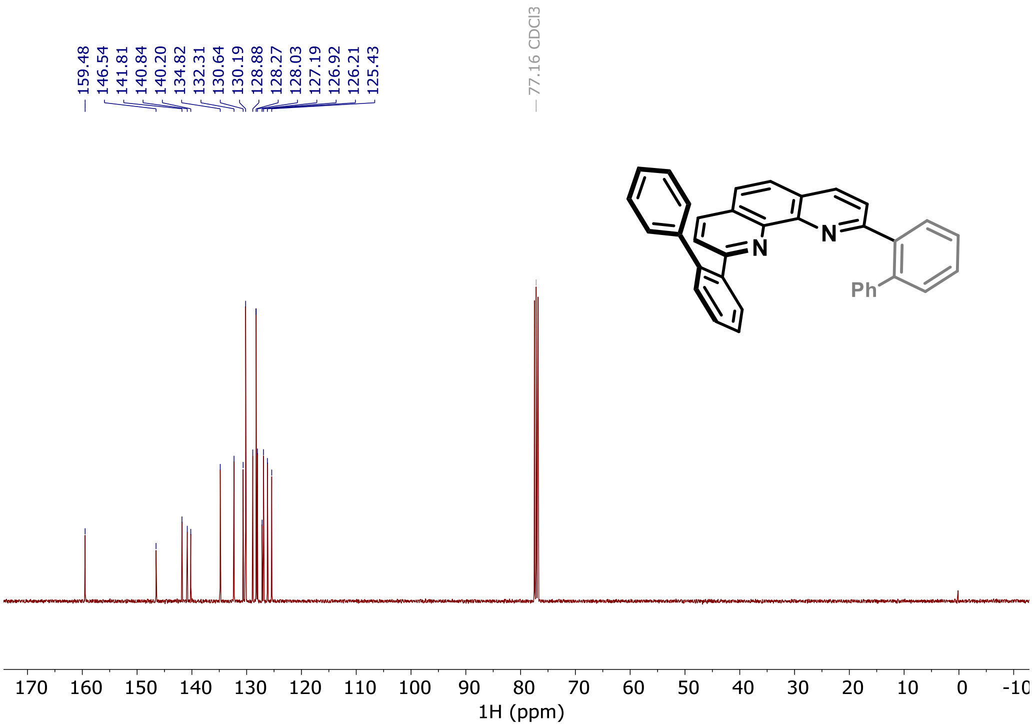

Complexes

A multipoint base multipoint baseline correction was conducted using the segments algorithm in MestReNova.

^{1}H NMR of 3 (400 MHz, $CDCl_3$, 25 °C)

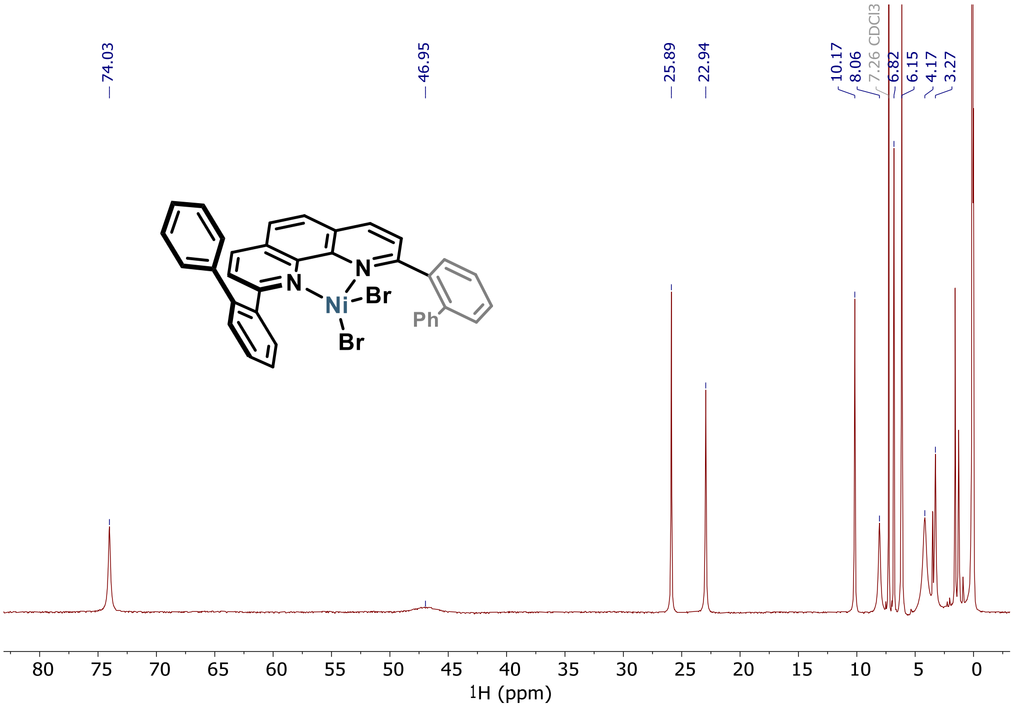

^{1}H NMR of 3 (400 MHz, THF-d_8, 25 °C)

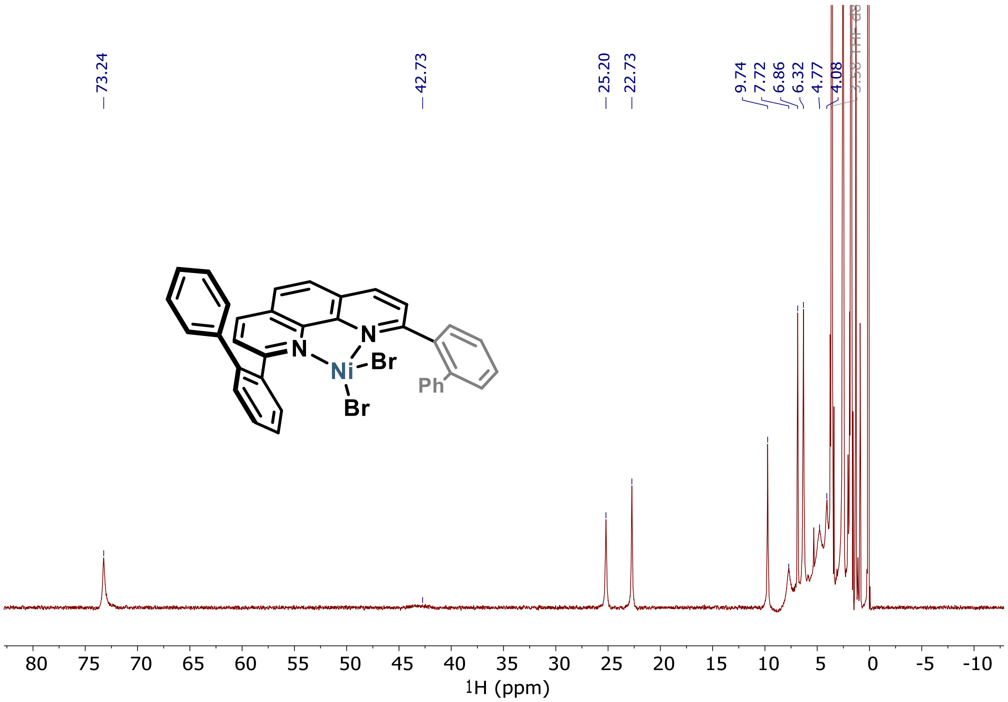

¹H NMR of 4 (400 MHz, THF-d_8, 25 °C)

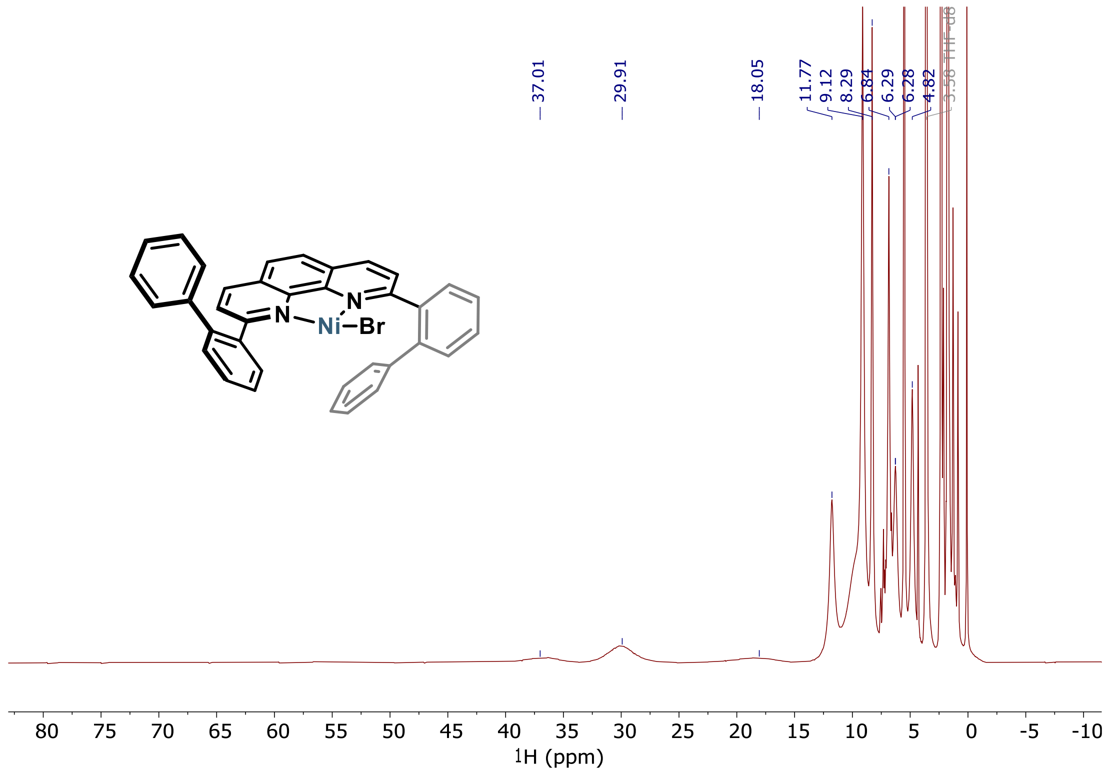

¹H NMR of 5 (400 MHz, THF-d_8, 25 °C)

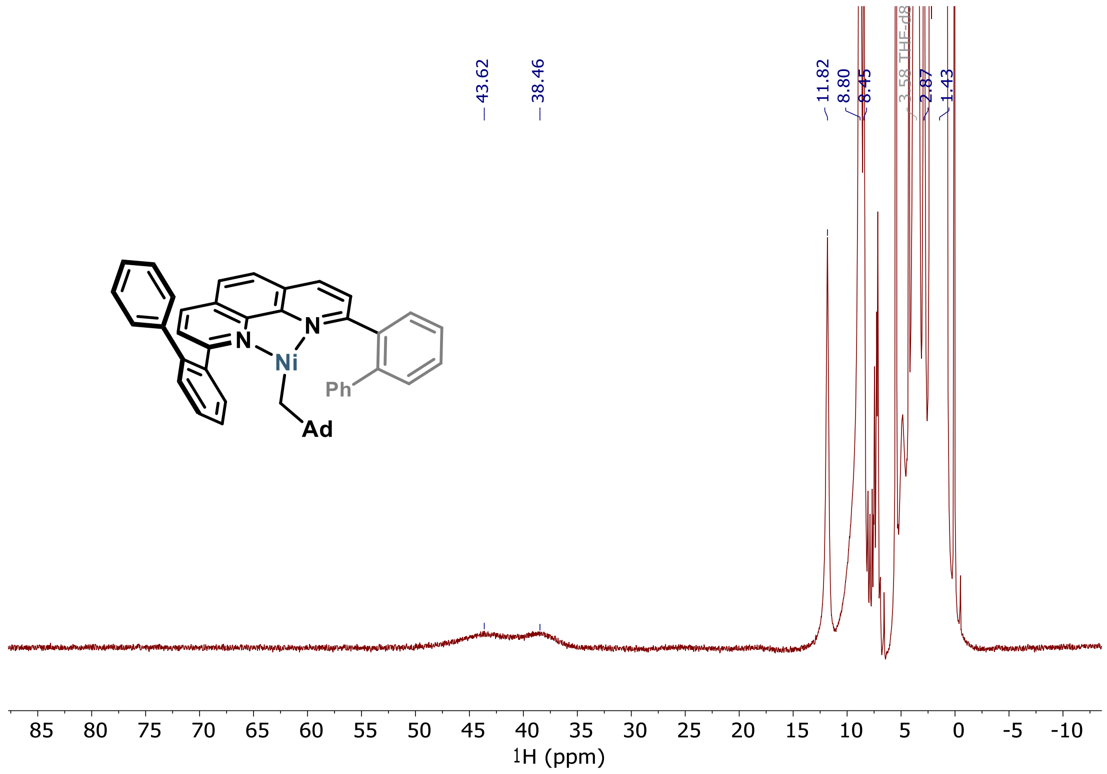

Supplement: Supplementary file 1 [file ja5c03351_si_001.pdf]
